# Macathiohydantoin *L*, a Novel Thiohydantoin Bearing a Thioxohexahydroimidazo [1,5-a] Pyridine Moiety from Maca (*Lepidium meyenii* Walp.)

**DOI:** 10.3390/molecules26164934

**Published:** 2021-08-14

**Authors:** Ranran Zhang, Junhong Liu, Hui Yan, Xingrong Peng, Ling Zhang, Minghua Qiu

**Affiliations:** 1 State Key Laboratory of Phytochemistry and Plant Resources in West China, Kunming Institute of Botany, Chinese Academy of Sciences, Kunming 650201, China; zhangranran@mail.kib.ac.cn (R.Z.); liujunhong@mail.kib.ac.cn (J.L.); yanhui@mail.kib.ac.cn (H.Y.); pengxingrong@mail.kib.ac.cn (X.P.); zhang-874005@163.com (L.Z.); 2 University of the Chinese Academy of Sciences, Beijing 100049, China

**Keywords:** *Lepidium meyenii*, thiohydantoins, thioxohexahydroimidazo [1,5-a] pyridine, neuroprotective activities

## Abstract

Five new thiohydantoin derivatives (**1**–**5**) were isolated from the rhizomes of *Lepidium meyenii* Walp. NMR (^1^H and ^13^C NMR, ^1^H−^1^H COSY, HSQC, and HMBC), HRESIMS, and ECD were employed for the structure elucidation of new compounds. Significantly, the structure of compound **1** was the first example of thiohydantoins with thioxohexahydroimidazo [1,5-a] pyridine moiety. Additionally, compounds **2** and **3** possess rare disulfide bonds. Except for compound **4**, all isolates were assessed for neuroprotective activities in corticosterone (CORT)-stimulated PC12 cell damage. Among them, compound (−)-**3** exhibited moderate neuroprotective activity (cell viability: 68.63%, 20 μM) compared to the positive control desipramine (DIM) (cell viability: 88.49%, 10 μM).

## 1. Introduction

Hydantoin, imidazolidine-2,4-dione, is a five-membered heterocycle that is one of the oxidized forms of imidazolidine with a cyclic urea core. The hydantoin scaffold has been enhanced in clinical use, for example, phenytoin, nitrofurantoin, and ethotoin. Thiohydantoin, an isosteric analogue of hydantoin, similarly possesses versatile biological activities, such as fungicidal, herbicidal [1], immunomodulating [2], and anticancer activities [3]. Based on enzalutamide, Xu et al. designed and synthesized a tetrahydroisoquinoline thiohydantoin scaffold. Several new analogues displayed improved antagonistic effect against the androgen receptor (AR) while maintaining the higher selective toxicity toward LNCaP cells (AR-rich) versus DU145 cells (AR-deficient) compared to enzalutamide [4]. However, (thio)hydantoin derivatives were rarely isolated from nature before 2017.

*Lepidium meyenii* Walp. (Brassicaceae), known as “Maca”, has been used as a traditional health care food for over 2000 years in South America. Modern pharmacological studies displayed its effects including strengthening body, improving fertility and sexual behavior [5,6], antioxidant [7], as well as anti-osteoporosis [8]. Recently, the potential neuroprotective activity of Maca has attracted a number of researchers [9,10,11]. Research has shown that extracts of Maca possessed effective neuroprotective activities in the 1-methyl-4-phenyl-1,2,3,6-tetrahydropyridine (MPTP)-induced zebrafish model [12].

The main chemical constituents of Maca are glucosinolates [13,14,15], macaenes, macamides [16,17,18,19,20], alkaloids [21,22,23,24], flavonols [25], phytosterols [14], polyscaccharides [26], and fatty acids. In our previous research, a series of pyrrole alkaloids [27] and thiohydantoin derivatives with cytotoxic and antimicrobial activities were found from Maca [28,29]. Recently, we consecutively isolated four pairs of unprecedented macathiohydantoin dimers, while (±) lepithiohyantoin B and (–) lepithiohyantoin D protected PC12 cells in a dose-dependent manner [30]. Notably, thiohydantoin and hydantoin derivatives isolated from the roots of Armoracia rusticana (Brassicaceae) exhibited potent nerve growth factor stimulation activities [31].

**Figure 1:**
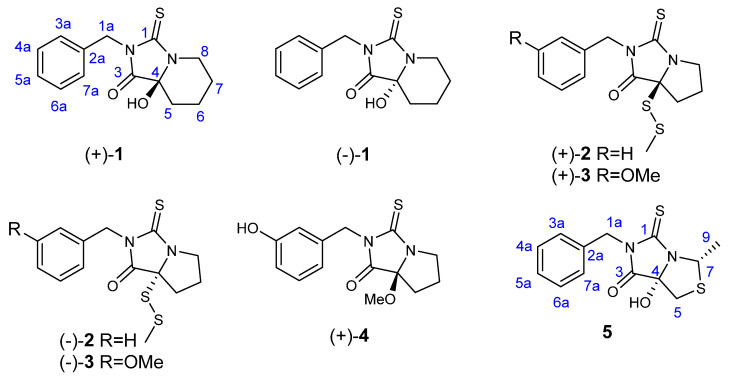
Structures of compounds **1**–**5**.

In the present work, we continued to investigate the constituents containing thiohydantoin moiety from Maca and five novel thiohydantoins, macathiohydantoins *l*−*O* (**1**–**4**) and (+)-Meyeniin d (**5**), were obtained from the rhizomes of Maca (Figure 1), of which compound **1** possesses thioxohexahydroimidazo [1,5-a] pyridine moiety. Additionally, compounds **2** and **3** possess rare disulfide bonds. Furthermore, their neuroprotective activities in PC12 cells induced by corticosterone (CORT) were evaluated.

## 2. Results and Discussion

### 2.1. Structure Determination of Macathiohydantoin L *(**1**)*

**Figure 2:**
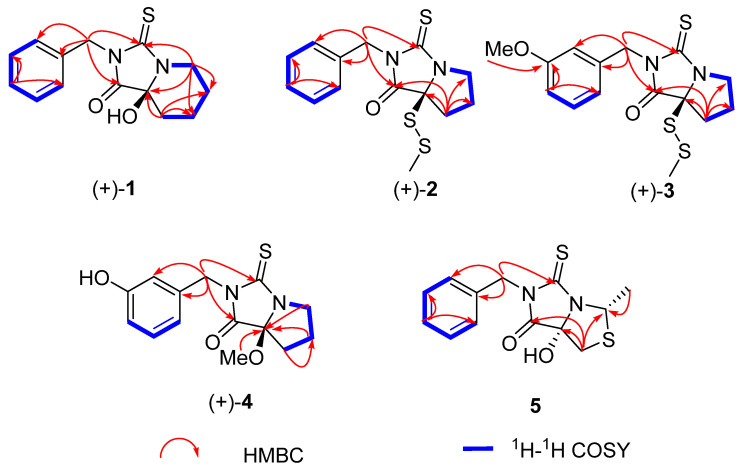
The key HMBC (^1^H→^13^C) and COSY (^1^H^1^H) correlations of compounds **1**–**5**.

Macathiohydantoin *L* (**1**) was isolated as yellow oil. The HRESIMS data gave an [M − H]^−^ ion at *m*/*z* 275.0866, which was consistent with a molecular formula of C_14_H_16_N_2_O_2_S and implied 8 indices of hydrogen deficiency. The ^13^C NMR data of **1** displayed characterized signals of two carbonyl groups (δ_C_ 179.2, 193.6), one monosubstituted phenyl ring (δ_C_ 135.8, 128.7 × 2, 128.5 × 2, 127.9) accounting for six degrees of unsaturation, and the remaining two ones indicated the presence of two rings in **1**. Comparing the 1D NMR data (supplementary materials) of macathiohydantoin *D* [29] and **1**, the presence of five methylenes (δ_C_ 18.4, 32.5, 24.7, 41.1, 44.7) were observed in **1** rather than four methylenes in macathiohydantoin *D*. The ^1^H−^1^H COSY correlations of H_2_-5-H_2_-6-H_2_-7-H_2_-8 and the HMBC correlations (Figure 2) of H_2_-8 with C-1, C-4, C-6, and C-7; and of δ_H_ 2.18 (1H, d, (*J* = 13.2, 2.4 Hz) H-5α) with C-4, C-6, and C-7 proved compound **1** was a thiohydantoin derivative with the thioxohexahydroimidazo [1,5-a] pyridine moiety.

**Figure 3:**
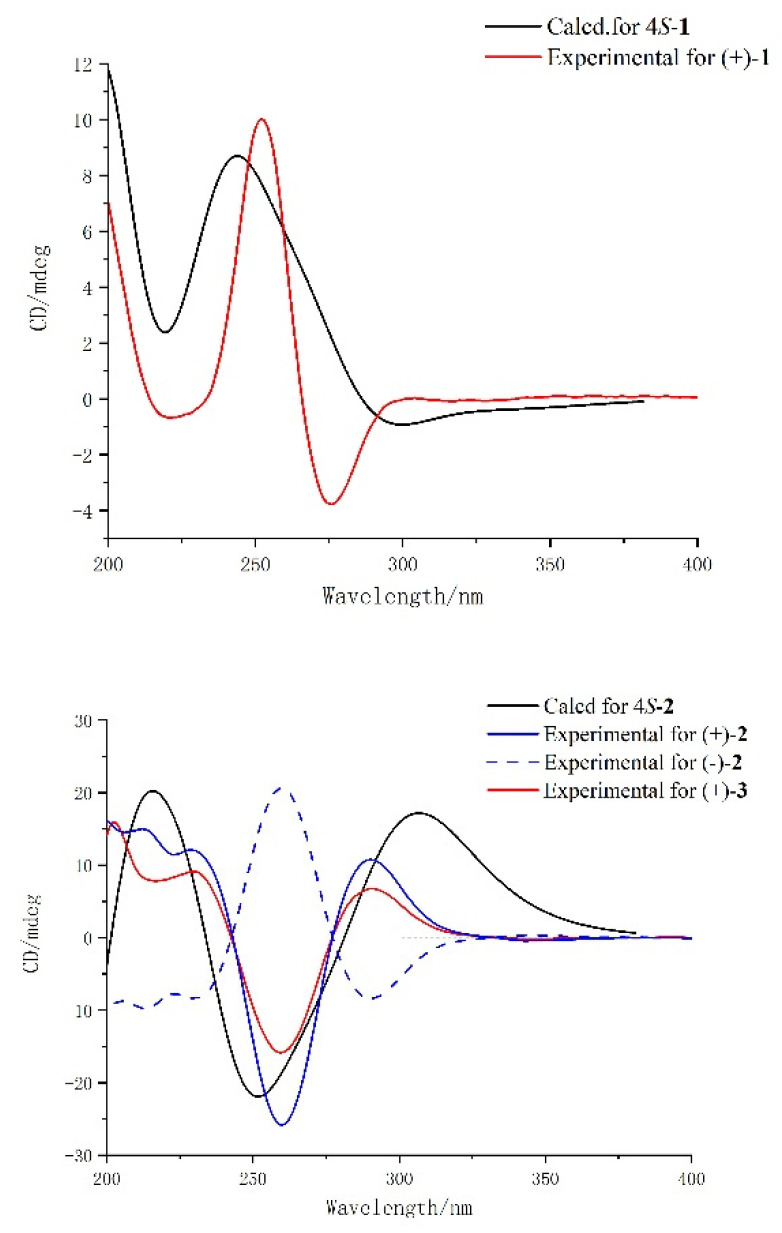

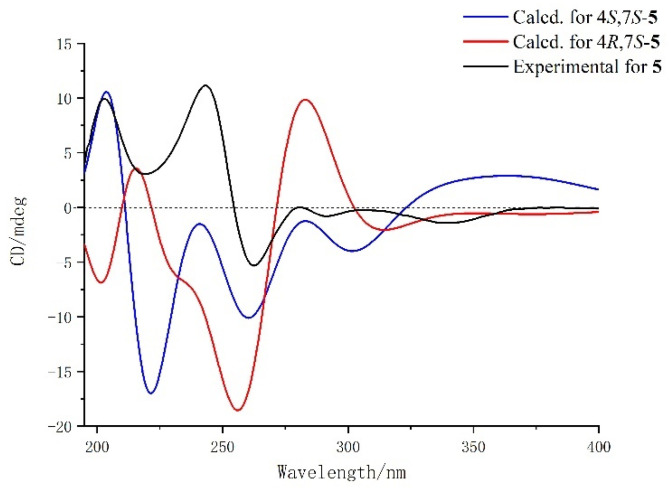
Experimental and calculated ECD spectra of compounds **1**, **2**, **3**, and **5**.

The specific rotation value [α${]}_{D}^{26}$ -7.07 (c 0.130, MeOH) of **1** suggested that it could be an enantiomer mixture, which was further substantiated by a chiral analysis. In order to determine the absolute configuration of enantiomers (+)-**1** and (–)-**1**, electronic circular dichroism (ECD) calculations were carried out. The predicted ECD spectrum of (4S)-**1** agreed well with the experimental CD spectrum of (+)-**1**, leading to the unambiguous assignment of the absolute configuration of 4*S* for (+)-**1** and 4*R* for (–)-**1**, respectively (Figure 3).

### 2.2. Structure Determination of Macathiohydantoin M *(**2**)*

Macathiohydantoin *M* (**2**) was isolated as colorless oil. The molecular formula of **2** was assigned as C_14_H_16_N_2_OS_3_ by HRESIMS data ([M + Na]^+^, *m*/*z* 347.0318, calcd 347.0317) with eight degrees of unsaturation. The ^1^H NMR spectrum (Table 1) of **2** displayed signals of five aromatic protons at δ_H_ 7.52 (2H, d, (*J* = 7.2 Hz), H-3a and H-7a), δ_H_ 7.26 (m, H-5a), and δ_H_ 7.30 (m, H-4a and H-6a) for monosubstituted phenyl moiety and one singlet methyl at δ_H_ 2.11 (s, H_3_-9). Additionally, four quaternary carbons (including two carbonyl groups) and four methylenes were assigned based on the ^13^C-DEPT spectra and the HSQC correlations. The aforementioned information showed that the structure of **2** was similar with that of macathiohydantoin *D* [29]. Simultaneously, the observed HMBC correlations (Figure 2) of H_2_-7 with C-1, C-4, C-5, and C-6; H_2_-5 with C-3, C-4, C-6, and C-7; and of H_2_-1a to C-1, C-3, C-2a, and C-3a, together with the ^1^H–^1^H COSY correlations of H_2_-5/H_2_-6/H_2_-7, further confirmed the above deduction. However, detailed comparison of their ^13^C NMR data displayed that the chemical shift of C-4 obviously shifted high-field in **2** (δ_C_ 80.3 for **2**, δ_C_ 92.5 for macathiohydantoin *D*). Considering two additional sulfur atoms and one singlet methyl in the molecular formula of **2**, a methyl disulfide bond was established and located at C-4.

Similarly, **2** was found to be also a pair of enantiomers through chiral analysis. The subsequent chiral HPLC resolution of **2** gave the anticipated enantiomers (–)-**2** and (+)-**2**, whose experimental CD curves were opposite. Thus, as depicted in Figure 3, the absolute configurations of (–)-**2** and (+)-**2** were deduced to be 4*R* and 4*S* by comparing with the calculated ECD curve of 4*S*-**2**.

### 2.3. Structure Determination of Macathiohydantoin N *(**3**)*

Macathiohydantoin *N* (**3**) exhibited a molecular formula of C_15_H_18_N_2_O_2_S_3_, as determined by HRESIMS at *m*/*z* 355.0600 [M + H]^+^ (calcd 355.0603). Inspection of the NMR data (Table 1) indicated a high similarity between **2** and **3**, except for an additional methoxyl and the replacement of monosubstituted phenyl by disubstituted phenyl in **2**. Further evidence was established from the HMBC correlations (Figure 2) of H_3_-OMe to C-4a and H_2_-1a to C-1, C-3, C-2a, C-3a.

Similarly, by comparison of experimental CD curves between (+)-**3** and (+)-**2**, the absolute configurations of (–)-**3** and (+)-**3** were determined as 4*R* and 4*S*, respectively.

### 2.4. Structure Determination of Macathiohydantoin O *(**4**)*

Macathiohydantoin *O* (**4**) was isolated as colorless oil with the molecular formula of C_14_H_16_N_2_O_3_S as deduced by HRESIMS data ([M − H]^−^, *m*/*z* 291.0818, calcd 291.0809). Compound **4** was also identified as a thiohydantoin derivative based on its 1D NMR data, which were similar with those of macathiohydantoin E [29] with the only difference in the methoxyl at C-4 in **4** instead of the hydroxyl in macathiohydantoin E. Furthermore, the HMBC correlation from H_3_-OMe to C-4 confirmed that methoxyl was located at C-4. Due to the specific rotation value of **4** being [α${]}_{D}^{26}$ +30.93 (c 0.120, MeOH) similar with (+)-macathiohydantoin *E* [+49.00 (c 0.007, MeOH)], the absolute configuration of (+)-**4** was directly deduced to be 4*S*.

### 2.5. Structure Determination of (+)-Meyeniin D *(**5**)*

(+)-Meyeniin *D* (**5**) as colorless powder was determined to be C_13_H_14_N_2_O_2_S_2_ based on the HRESIMS data observed at *m*/*z* 293.0426 [M − H]^−^, (calcd for C_13_H_13_N_2_O_2_S_2_, 293.0424). Its 1D NMR spectroscopic data were similar with (+)-meyeniins *B* [32] except that H-4 in (+)-meyeniins *B* was replaced by a hydroxy group. The inference was further proved by the HMBC correlations of δ_H_ 1.73 (3H, d, (*J* = 6.4 Hz), H-9) with C-7, H_2_-5 with C-7, C-4, and C-3, and H_2_-1a with C-1, C-3, C-2a, and C-3a. The absolute configuration of **5** was determined as (4*S*, 7*S*) by ECD calculations (Figure 3).

### 2.6. Neuroprotective Activities of Selected Compounds

Except for compound **4**, all isolates were assessed for their neuroprotective activities in corticosterone (CORT)-stimulated poorly differentiated PC12 cells. Compound(–)-**3** exhibited the most potent neuroprotective activity (cell viability: 68.63%, 20 μM). Interestingly, the compounds **1**–**3** with 4S-configuration showed higher activities compared to their enantiomers (Table 2).

## 3. Materials and Methods

### 3.1. General Experimental Procedures

Optical rotations were obtained with a Rudolph Autopol VI polarimeter in MeOH. A Shimadzu UV-2700 spectrometer was used to obtain UV spectra. ^1^H and ^13^C NMR spectra were acquired on Bruker AV-600 and AV-800 instruments (Bruker, Zurich, Switzerland) using tetramethylsilane (TMS) as an internal standard for chemical shifts in CDCl_3_. Chemical shifts (*δ*) were expressed in ppm and referenced to the TMS resonance. High-resolution electrospray ionization mass spectrometry (HRESIMS) data were performed on an UPLC system (1260, Agilent) coupled to a quadrupole time-of-flight mass spectrometer (Agilent 6540 Q-TOF, Agilent Technologies, Foster City, CA, USA). Infrared spectra were recorded on a Bruker Tensor-27 instrument by using KBr pellets. An Agilent 1100 series instrument equipped with an Agilent ZORBAX SB-C18 column (5 μm, 9.4 mm × 250 mm) was used for high-performance liquid chromatography (HPLC) analysis. Chiral chromatography using a CHIRALCEL AD-H column (5 μm, 4.6 mm × 150 mm) was used to resolve enantiomers.

Silica gel (200–300 mesh, Qingdao Marine Chemical, Inc.), Lichroprep RP-18 (40–63 μm, Merck), and Sephadex LH-20 (20–150 μm, Pharmacia, Sweden) were used for column chromatography. Fractions were monitored by TLC (GF254, Qingdao Marine Chemical Ltd., Qingdao, China) and by heating silica gel plates sprayed with 10% H_2_SO_4_ in ethanol. Methanol, dichloromethane, ethylacetate, acetone, and petroleum ether were purchased from Yunnan Chemical Reagent Co. (Yunann, China). All other materials were of the highest grade available.

### 3.2. Plant Material 

Rhizomes of Maca (*Lepidium meyenii* Walp.) purchased in September 2019 from a Luo-shiwan Traditional Chinese Medicine Market in Kunming were collected from Lijiang of Yunnan, China. Maca was identified by Prof. Qiu Minghua, who works at Kunming Institute of Botany, Chinese Academy of Sciences. The specimen was kept in the State Key Laboratory of Phytochemistry and Plant Resources in West China, Kunming Institute of Botany, Chinese Academy of Sciences, Kunming of China.

### 3.3. Plant Material Extraction and Isolation

The air-dried and powered maca rhizomes (37 kg) were extracted three times with acetone at room temperature and evaporated to remove solutions to yield the crude extract. The aqueous residue was extracted with petroleum ether (PE, I) and ethyl acetate (EtOAc, II), respectively.

The PE part (267 g) was subjected to a silica gel column with PE/ EtOAc (50:1→1:1, *v*/*v*) to yield seven fractions (Fr. I-1–Fr. I-7). Fr. I-2 (15 g) was further subjected to an RP-C18 column with MeOH/H_2_O (40:60→100:0, *v*/*v*) to afford four subfractions (Fr. I-2-1–Fr. I-2-4). Fr. I-2-3 (65 mg) was separated by a Sephadex LH-20 column (MeOH) to afford compounds **2** (11.9 mg) and **1** (2.2 mg). Similarly, Fr. I-3 (22 g) was also separated with a RP-18 column with MeOH/H_2_O (40:60→100:0, *v*/*v*) to afford four subfractions (Fr. I-3-1–Fr. I-3-4). Fr. I-3-4 was subjected to a Sephadex LH-20 column (MeOH) to afford four subfractions (Fr. I-3-4-1–Fr. I-3-4-4). Semi-preparative HPLC afforded compounds **3** (3.5 mg) in Fr. I-3-4-3, and compound **4** (0.8 mg), **5** (1.6 mg) were isolated from Fr. I-4-2 in the same way.

Compounds **1**‒**3** were respectively separated by chiral analytic column to get (+)-**1** (1.9 mg, t*_R_* = 9.3 min) and (–)-**1** (0.9 mg, t*_R_* = 10.5 min) (AD-H, n-hexane/isopropanol = 92:8, *v*/*v*, flow rate = 1.0 mL/min); (+)-**2** (1.8 mg, t*_R_* = 17.0 min) and (–)-**2** (1.8 mg, t*_R_* = 20.1 min) (AD-H, n-hexane/isopropanol = 92:8, *v*/*v*, flow rate = 1.0 mL/min); (+)-**3** (1.5 mg, t*_R_* = 15.0 min) and (–)-**3** (1 mg, t*_R_* = 18.7 min) (AD-H, n-hexane/isopropanol = 92:8, *v*/*v*, flow rate = 1.0 mL/min).

#### 3.3.1. Macathiohydantoin L (**1**)

Yellow oil (MeOH); [α${]}_{D}^{26}$ − 7.07 (c 0.130, MeOH); {(+)-**1**: [α${]}_{D}^{16}$ + 25.43 (c 0.190, MeOH); CD (MeOH) Δε215 − 0.21, Δε250 + 9.70, Δε272 − 3.30, Δε291 − 0.68; (–)-**1**: [α${]}_{D}^{16}$ − 16.02 (c 0.090, MeOH); CD (MeOH) Δε215 + 0.97, Δε250 − 0.48, Δε271 + 0.64, Δε303 + 0.18}; UV (MeOH) *λ*_max_ (log ε): 283 (4.69), 261 (4.72), 275 (4.68), and 233 (4.40) nm; ^1^H NMR and ^13^C NMR data: see Table 1; IR (KBr) *ν*_max_ 3832, 2926, 2854, 1751, 1641, 1481, 1439, and 1361 cm^−1^; HRESIMS *m*/*z* 275.0866 [M − H] ^−^ (calcd for C_14_H_15_N_2_O_2_S, 275.0860).

#### 3.3.2. Macathiohydantoin M (**2**)

Colorless oil (MeOH); [α${]}_{D}^{26}$ + 8.89 (c 0.140, MeOH); {(+)-**2**: [α${]}_{D}^{26}$ + 24.04 (c 0.190, MeOH); CD (MeOH) Δε 201 + 15.79, Δε 257 − 24.69, Δε 280 + 4.32, Δε 303 + 5.59; (–)-**2**: [α${]}_{D}^{26}$ − 10.63 (c 0.160, MeOH); CD (MeOH) Δε201 − 9.33, Δε257 + 19.86, Δε280 − 3.29, Δε303 − 4.37}; UV (MeOH) *λ*_max_ (log ε): 283 (4.16), 262 (4.14), 271 (4.13), and 230 (3.72) nm; ^1^H NMR and ^13^C NMR data: see Table 1; IR (KBr) *ν*_max_ 2924, 2854, 1746, 1605, 1586, 1419, 1372, and 1242 cm^−1^; HRESIMS *m*/*z* 347.0318 [M + Na]^+^ (calcd for C_14_H_16_N_2_OS_3_Na, 347.0317).

#### 3.3.3. Macathiohydantoin N (**3**)

Colorless oil (MeOH); [α${]}_{D}^{26}$ + 4.92 (c 0.130, MeOH); {(+)-**3**: [α${]}_{D}^{24}$ + 37.38 (c 0.080, MeOH); CD (MeOH) Δε201 + 15.32, Δε257 − 15.34, Δε280 + 2.78, Δε303 + 3.65; (–)-**3**: [α${]}_{D}^{25}$ − 38.44 (c 0.050, MeOH); CD (MeOH) Δε201 − 12.91, Δε257 + 16.33, Δε280 − 2.87, Δε303 − 3.78}; UV (MeOH) *λ*_max_ (log ε): 279 (4.27), 237 (4.02), and 196 (4.84) nm; ^1^H NMR and ^13^C NMR data: see Table 1; IR (KBr) *ν*_max_ 2924, 2852, 1747, 1602, 1587, 1417, 1342, and 1239 cm^−1^; HRESIMS *m*/*z* 355.0600 [M + H]^+^ (calcd for C_15_H_19_N_2_O_2_S_3_, 355.0603).

#### 3.3.4. Macathiohydantoin O (**4**)

Colorless oil (MeOH); [α${]}_{D}^{26}$ + 30.93 (c 0.120, MeOH); UV (MeOH) *λ*_max_ (log ε): 271 (3.50), 234 (3.20), and 197 (3.92) nm; ^1^H NMR and ^13^C NMR data: see Table 1; IR (KBr) *ν*_max_ 3429, 2919, 2850, 1754, 1591, 1423, and 1259 cm^−1^; HRESIMS *m*/*z* 291.0818 [M − H] ^−^ (calcd for C_14_H_15_N_2_O_3_S, 291.0809).

#### 3.3.5. (+)-Meyeniin D (**5**)

Colorless oil (MeOH); [α${]}_{D}^{26}$ + 108.38 (c 0.08, MeOH); CD (MeOH) Δε201 + 9.54, Δε241 − 10.74, Δε260 − 4.82, Δε278 − 0.22; UV (MeOH) *λ*_max_ (log ε): 272 (3.96), 231 (3.61), and 196 (4.24) nm; ^1^H NMR and ^13^C NMR data: see Table 1; IR (KBr) *ν*_max_ 2926, 2853, 1756, 1606, 1414, 1383, and 1194 cm^−1^; HRESIMS *m*/*z* 293.0426 [M − H] ^−^ (calcd for C_13_H_13_N_2_O_2_S_2_, 293.0424).

### 3.4. Cell Culture and Cell Viability Assays

Poorly differentiated PC12 cells were maintained in Dulbecco’s modified eagle medium (DMEM) supplemented with 10% fetal bovine serum (FBS), penicillin (100 U/mL), streptomycin (100 μg/mL), and incubated at 5% CO_2_ and 37 °C. Poorly differentiated PC12 cells were divided into the following groups: untreated, CORT (150 μmol/L), CORT (150 μmol/L) plus DIM (10 μmol/L), CORT (150 μmol/L) plus test compounds (20 μmol/L). Briefly, poorly differentiated PC12 cells were seeded into 96-well culture plates at a density of 1*104 cells/well. After 24 h culturing, the wells were added compounds as previously described groups. Then, 48 h later, MTS solution was added to each well. The absorbance was measured at 492 nm using a Thermo Multiskan FC. 

## 4. Conclusions

In summary, five new thiohydantoin derivatives (**1**‒**5**) were isolated from the rhizomes of *L. meyenii*. Specifically, compound **1** possesses thioxohexahydroimidazo [1,5-a] pyridine moiety. Additionally, compounds **2** and **3** possess the rare disulfide bonds, and compound (–)-**3** exhibited moderate neuroprotective activity compared with desipramine (DIM) as a positive control. Our research not only enriches the structural types of compounds in Maca but also provides a material basis for Maca as a potential health food to treat neurodegenerative diseases.

## Figures and Tables

**Table 1:** ^1^H NMR, ^13^C NMR, and DEPT spectroscopic data of compounds **1**–**5** in CDCl_3_.

	1 ^a^		2 ^a^		3 ^a^		4 ^b^		5 ^a^	
	*δ*_H_	*δ*_C_	*δ*_H_	*δ*_C_	*δ*_H_	*δ*_C_	*δ*_H_	*δ*_C_	*δ*_H_	*δ*_C_
1		179.2s		184.6 s		184.6 s		186.2 s		182.8 s
3		173.6 s		173.1 s		173.1 s		170.2 s		169.4 s
4		83.1 s		80.3 s		80.4 s		97.2 s		93.8 s
5	2.18, d (14.0) 1.47, m	32.5 t	2.15, m	31.6 t	2.17, m 2.37,m	31.6t	2.19, m 1.75, m	32.4 t	3.21, d (12.0) 3.06, d (12.0)	38.4 t
6	1.97 m 1.81 m	18.4 t	2.35, m 2.23, m	25.7 t	2.23, m 2.17, m	25.7 t	2.36, m 2.19, m	24.9 t		
7	1.82, m 1.50, m	24.7 t	4.10, m 3.67, m	47.4 t	4.09, m 3.66, m	47.4 t	4.05, dt (10.8, 8.4) 3.59, ddd (10.8, 9.0, 3.0)	48.0 t	5.60, q (6.4)	61.0 d
8	4.68, dd (13.3,4.7) 3.30, dd (13.2,2.4)	41.1 t								
9			2.11, s	23.2 q	2.15, s	23.3 q			1.73, d (6.4)	24.5 q
1a	5.05, d (14.5) 5.00, d (14.5)	44.7 t	5.15, d (14.5) 4.91, d (14.5)	45.4 t	5.10, d (14.5) 4.89, d (14.5)	45.4 t	4.98, d(14.4) 4.91, d (14.4)	44.8 t	4.98, d (14.5) 4.87, d (14.5)	45.5 t
2a		135.8 s		135.5 s		136.9 s		137.3 s		135.3 s
3a	7.44, d (7.3)	128.7 d	7.52, d (7.2)	128.8 d	7.09, m	114.0 d	6.93, s	115.5 d	7.43, d (7.2)	129.0 d
4a	7.30, m	128.5 d	7.30, m	128.4 d		159.6 s		155.7 s	7.32, m	128.9 d
5a	7.29, m	127.9 d	7.26, m	127.9 d	6.81, d (6.0)	113.6 d	6.75, d (7.8)	115.0 d	7.32, m	128.4 d
6a	7.30, m	128.5 d	7.30, m	128.4 d	7.22, t (8.1)	129.9 d	7.17, t (7.8)	129.9 d	7.32, m	128.9 d
7a	7.44, d (7.3)	128.7 d	7.52, d (7.2)	128.8 d	7.09, m	121.1 d	7.00, d (7.8)	121.0 d	7.43, d (7.2)	129.0 d
OMe					2.81, s	55.2 q	3.13, s	51.9 q		

^a^ Measured at 600/150 MHz; ^b^ Measured at 800/200 MHz.

**Table 2:** Neuroprotective activities of selected compounds.

Compound	Concentration (μmol)	Cell Viability (%)
DIM ^a^	10	88.49 ± 1.49
(+)-**1**	20	60.37 ± 0.29
(−)-**1**	20	62.59 ± 0.36
(+)-**2**	20	65.85 ± 1.35
(−)-**2**	20	67.64 ± 2.88
(+)-**3**	20	65.60 ± 1.18
(−)-**3**	20	68.63 ± 1.12
**5**	20	63.32 ± 1.10

^a^ Positive control substance. Results are the means of three independent experiments, and the data are expressed as mean ± SD.

## Data Availability

All data are available in this publication and in the Supplementary Materials.

## Supplementary Materials

The following are available online. 1D and 2D NMR spectra of all isolated compounds. Detailed information for each material is given in the Supplementary Material.

## Sample Availability

Samples of the compounds are not available from the authors.
