# Supplementary material for: Macathiohydantoin L, a Novel Thiohydantoin Bearing a Thioxohexahydroimidazo [1,5-a] Pyridine Moiety from Maca (Lepidium meyenii Walp.)
Source: Molecules. 2021 Aug 14;26(16):4934. doi: 10.3390/molecules26164934 (PMC8398295; doi:10.3390/molecules26164934)
Supplement: Supplementary file 1 [file molecules-26-04934-s001.zip › molecules-1333029-supplementary.pdf]

Macathiohydantoin *L*, a Novel Thioxohexahydroimidazo [1,5-a] Pyridine Derivatives from Maca (*Lepidium meyenii*)

Ranran Zhang [1, 2], Junhong Liu [1,2], Hui Yan[1], Xingrong Peng [1], Ling Zhang[1] and Minghua Qiu[1, *]

*[1]*State Key Laboratory of Phytochemistry and Plant Resources in West China, Kunming Institute of Botany, Chinese Academy of Science, Kunming 650201, People's Republic of China*

Email: zhangranran@mail.kib.ac.cn. Email: liujunhong@mail.kib.ac.cn. Email: yanhui@mail.kib.ac.cn. Email: pengxingrong@mail.kib.ac.cn Email: zhang-874005@163.com

[2] *University of the Chinese Academy of Science, Beijing 100049, People's Republic of China*

* *Corresponding author: Telephone: +86-0871-65223327. Fax: +86-0871-65223325. E-mail: mhchiu@mail.kib.ac.cn*

Table of Contents

Figure S1-1. HR-ESI-MS spectrum of **1** 5

Figure S1-2 ESI-MS spectrum of **1** 6

Figure S1-3 The chiral analytical HPLC chromatography of **1**..................6

Figure S1-4. [α] $_D$ data of (+)-**1** 6

Figure S1-5. [α] $_D$ data of (–)-**1** 7

Figure S1-6. CD and UV spectrum of (+)-**1** in MeOH...............................8

Figure S1-7. CD and UV spectrum of (–)-**1** in MeOH...............................9

Figure S1-8. ^{1}H NMR spectrum of **1** in $CDCl_3$ (600 MHz).....................12

Figure S1-9. ^{13}C NMR spectrum of **1** in $CDCl_3$ (150 MHz)....................12

Figure S1-10. ^{1}H-^{1}H COSY spectrum of **1** in $CDCl_3$ (600 MHz)...........13

Figure S1-11. HSQC spectrum of **1** in $CDCl_3$ (600 MHz).......................13

Figure S1-12 HMBC spectrum of **1** in $CDCl_3$ (600 MHz).......................14

Figure S2-1. HR-ESI-MS spectrum of **2** 15

Figure S2-2 ESI-MS spectrum of **2** 16

Figure S2-3 The chiral analytical HPLC chromatography of **2**................16

Figure S2-4. [α] $_D$ data of (+)-**2** 17

Figure S2-5. [α] $_D$ data of (–)-**2** 17

Figure S2-6. CD and UV spectrum of (+)-**2** in MeOH.............................18

Figure S2-7. CD and UV spectrum of (–)-**2** in MeOH.............................19

Figure S2-8. ^{1}H NMR spectrum of **2** in $CDCl_3$ (600 MHz) 22

Figure S2-9. ^{13}C NMR spectrum of **2** in $CDCl_3$ (150 MHz) 22

Figure S2-10. ^{1}H-^{1}H COSY spectrum of **2** in $CDCl_3$ (600 MHz) 23

Figure S2-11. HSQC spectrum of **2** in $CDCl_3$ (600 MHz).......................23

Figure S2-12 HMBC spectrum of **2** in $CDCl_3$ (600 MHz).......................24

Figure S3-1. HR-ESI-MS spectrum of **3** 25

Figure S3-2. ESI-MS spectrum of **3** 26

Figure S3-2 The chiral analytical HPLC chromatography of **3**...............26

Figure S3-3. [α] D data of (+)-**3** 26

Figure S3-4. [α] $_{D}$ data of (–)-**3** 27

Figure S3-5. CD and UV spectrum of (+)-**3** in MeOH............................27

Figure S3-6. CD and UV spectrum of (–)-**3** in MeOH............................28

Figure S3-7. ^{1}H NMR spectrum of **3** in $CDCl_3$ (600 MHz) 30

Figure S3-8. ^{13}C NMR spectrum of **3** in $CDCl_3$ (150 MHz) 30

Figure S3-9. ^{1}H-^{1}H COSY spectrum of **3** in $CDCl_3$ (600 MHz) 31

Figure S3-10. HSQC spectrum of **3** in $CDCl_3$ (600 MHz).......................31

Figure S3-11 HMBC spectrum of **3** in $CDCl_3$ (600 MHz).......................32

Figure S4-1. HR-ESI-MS spectrum of **4** 33

Figure S4-2. ESI-MS spectrum of **4** 34

Figure S4-3 UV spectrum of **4** in MeOH 35

Figure S4-4 [α] $_{D}$ data of **4** 36

Figure S4-5 ^{1}H NMR spectrum of **4** in $CDCl_3$ (600 MHz) 36

Figure S4-6 ^{13}C NMR spectrum of **4** in $CDCl_3$ (150 MHz) 37

Figure S4-7 ^{1}H-^{1}H COSY spectrum of **4** in $CDCl_3$ (600 MHz) 37

Figure S4-8 HSQC spectrum of **4** in $CDCl_3$ (600 MHz)..........................38

Figure S4-9 HMBC spectrum of **4** in $CDCl_3$ (600 MHz).........................38

Figure S5-1. HR-ESI-MS spectrum of 5 39

Figure S5-2. ESI-MS spectrum of **5** 40

Figure S5-3 $[\alpha]_D$ data of **5** 40

Figure S5-4. CD and UV spectrum of **5** in MeOH 41

Figure S5-5. ^{1}H NMR spectrum of 5 in $CDCl_3$ (600 MHz) 43

Figure S5-6. ^{13}C NMR spectrum of **5** in $CDCl_3$ (150 MHz) 43

Figure S5-7. ^{1}H-^{1}H COSY spectrum of **5** in $CDCl_3$ (600 MHz) 44

Figure S5-8. HSQC spectrum of **5** in $CDCl_3$ (600 MHz)........................44

Figure S5-9. HMBC spectrum of **5** in $CDCl_3$ (600 MHz).......................44

Figure S6 Neuroprotective activities of selected compounds 45

Supporting information list

Figure S1-1. HR-ESI-MS spectrum of **1**

Qualitative Analysis Report

**Data Filename**	kmch-13.d	**Sample Name**	kmch-13
**Sample Type**	Sample	**Position**	P1-A1
**Instrument Name**	Instrument 1	**User Name**	
**Acq Method**	s-.m	**Acquired Time**	11/27/2020 2:06:39 PM
**IRM Calibration Status**	Success	**DA Method**	Default.m
**Comment**			

Sample Group **Info.**
Acquisition SW 6200 series TOF/6500 series
Version Q-TOF B.05.01 (B5125.2)

User Spectra

Fragmentor Voltage	Collision Energy	Ionization Mode
175	0	ESI

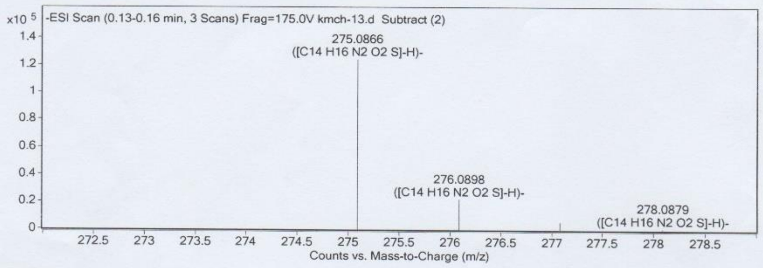

Peak List

m/z	z	Abund	Formula	Ion
57.9759		6669.81		
98.0612	1	18541.84		
126.0559	1	34674.01		
132.0455	1	6895.82		
148.0225	1	21276.3		
247.0913	1	17247.96		
275.0866	1	124234.81	C14 H16 N2 O2 S	(M-H)-
276.0898	1	21887.83	C14 H16 N2 O2 S	(M-H)-
305.0972	1	9340.53		
551.18	1	7666.75		

Formula Calculator Element Limits

Element	Min	Max
C	14	14
H	0	120
O	0	30
N	0	10
S	0	10

Formula Calculator Results

Formula	CalculatedMass	CalculatedMz	Mz	Diff. (mDa)	Diff. (ppm)	DBE
C14 H16 N2 O2 S	276.0933	275.0860	275.0866	-0.60	-2.18	8.0000

--- End Of Report ---

Agilent Technologies Page 1 of 1 Printed at: 2:10 PM on: 11/27/2020

Figure S1-2 ESI-MS spectrum of **1**

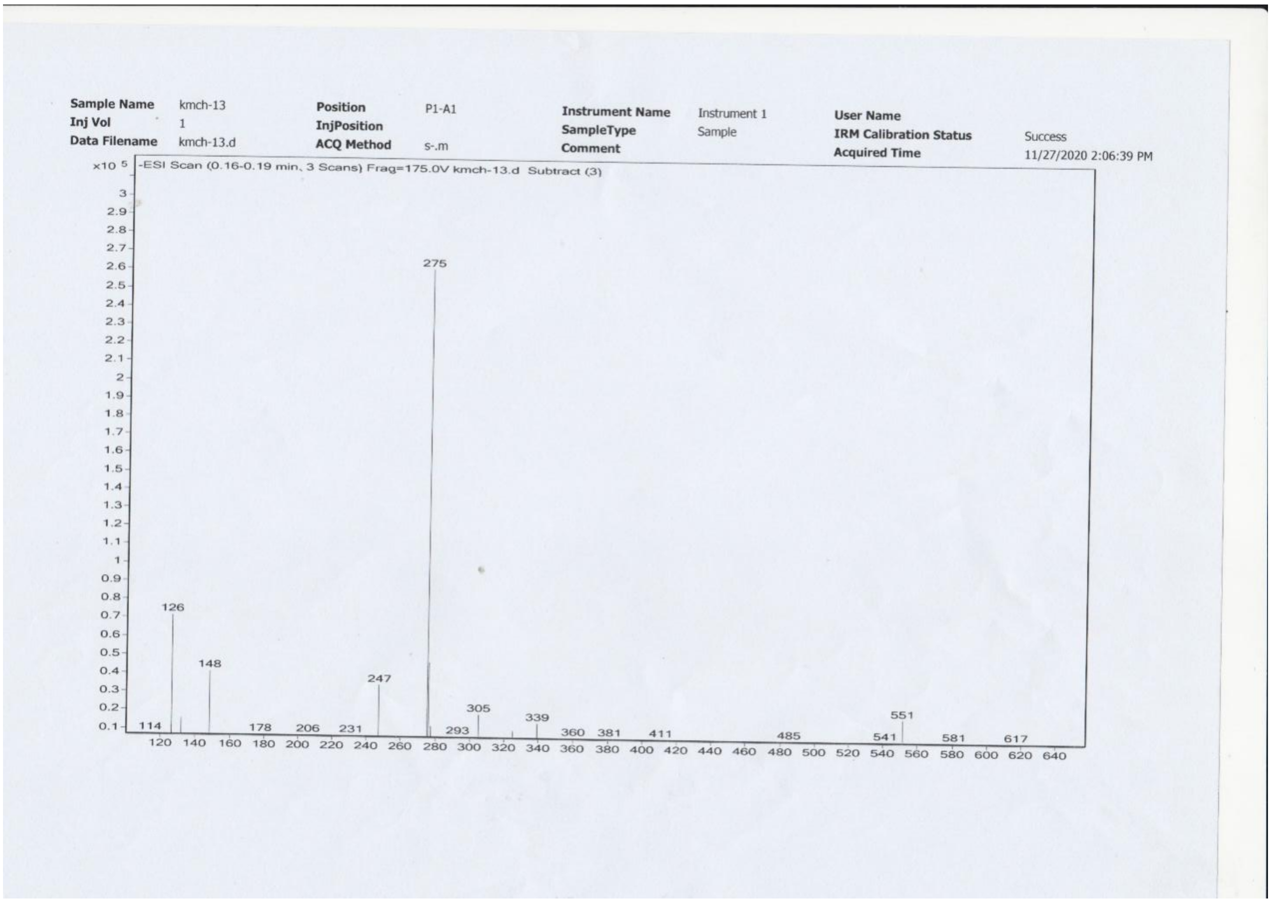

Figure S1-3 The chiral analytical HPLC chromatography of **1**

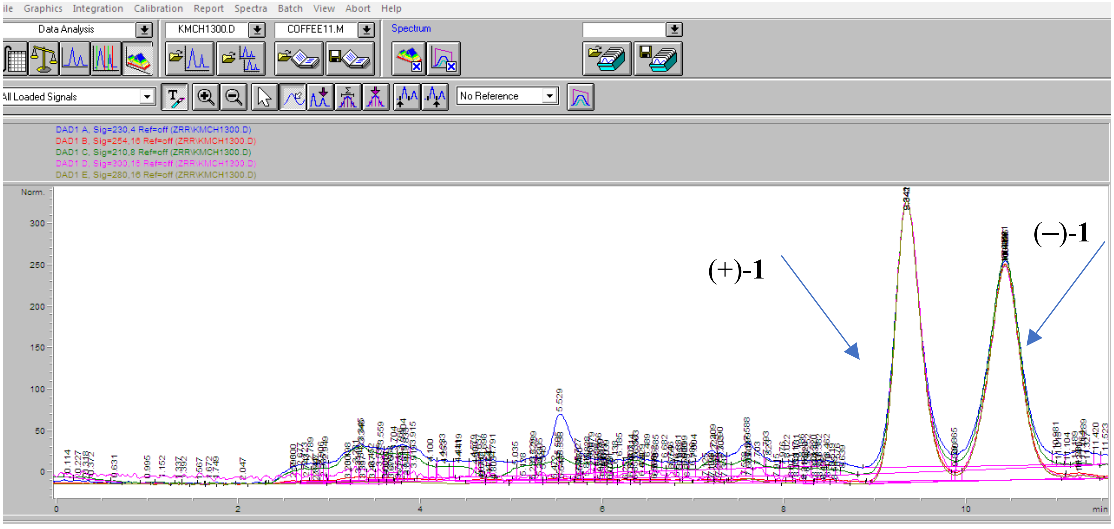

Figure S1-4. [α] D data of (+)-**1**

Rudolph Research Analytical

This sample was measured on an Autopol VI, Serial #91058
Manufactured by Rudolph Research Analytical, Hackettstown, NJ, USA.

Measurement Date : Monday, 11-JAN-2021

Set Temperature : OFF

Time Delay : Disabled

Delay between Measurement : Disabled

n	Average	Std.Dev.	% RSD	Maximum	Minimum
5	25.43	0.13	0.51	25.63	25.32

S.No	Sample ID	Time	Result	Scale	OR °Arc	WLG.nm	Lg.mm	Conc.g/100ml	Temp.
1	KMCH-13-1	02:22:40 PM	25.42	SR	0.0483	589	100.00	0.190	16.2
2	KMCH-13-1	02:22:48 PM	25.47	SR	0.0484	589	100.00	0.190	16.2
3	KMCH-13-1	02:22:56 PM	25.32	SR	0.0481	589	100.00	0.190	16.2
4	KMCH-13-1	02:23:05 PM	25.63	SR	0.0487	589	100.00	0.190	16.2
5	KMCH-13-1	02:23:13 PM	25.32	SR	0.0481	589	100.00	0.190	16.2

Figure S1-5. [α] $_D$ data of (−)-**1**

Rudolph Research Analytical

This sample was measured on an Autopol VI, Serial #91058
Manufactured by Rudolph Research Analytical, Hackettstown, NJ, USA.

Measurement Date : Monday, 11-JAN-2021

Set Temperature : OFF

Time Delay : Disabled

Delay between Measurement : Disabled

n	Average	Std.Dev.	% RSD	Maximum	Minimum
5	-16.02	0.14	-0.87	-15.89	-16.22

S.No	Sample ID	Time	Result	Scale	OR °Arc	WLG.nm	Lg.mm	Conc.g/100ml	Temp.
1	KMCH-13-2	02:35:46 PM	-16.11	SR	-0.0145	589	100.00	0.090	16.0
2	KMCH-13-2	02:35:54 PM	-16.22	SR	-0.0146	589	100.00	0.090	16.0
3	KMCH-13-2	02:36:02 PM	-15.89	SR	-0.0143	589	100.00	0.090	16.0
4	KMCH-13-2	02:36:11 PM	-16.00	SR	-0.0144	589	100.00	0.090	16.0
5	KMCH-13-2	02:36:19 PM	-15.89	SR	-0.0143	589	100.00	0.090	16.0

Figure S1-6. CD and UV spectrum of (+)-**1** in MeOH

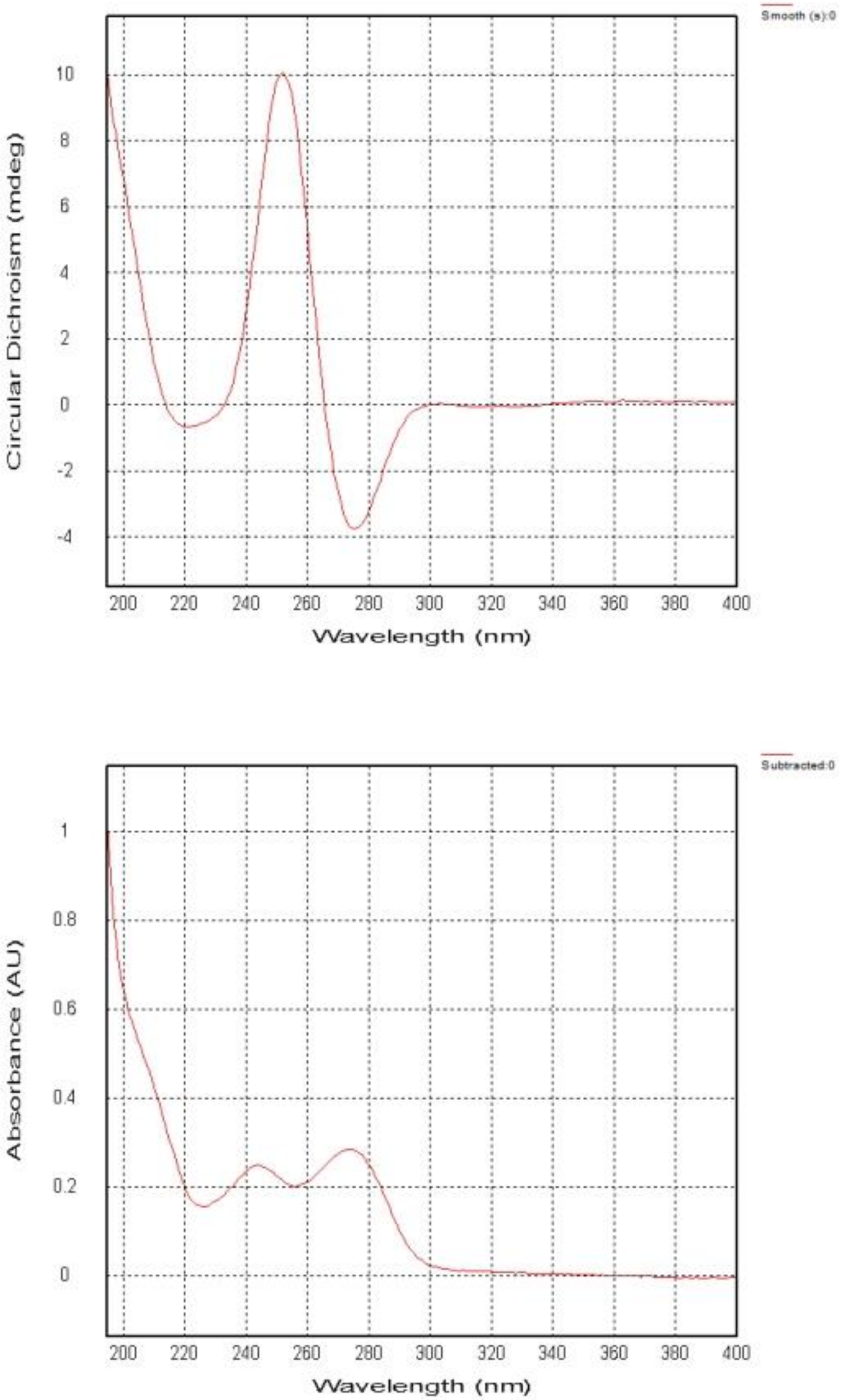

File: KMCH-13-1-1mm (195-400 nm) 20122305.dsx
ProBinaryX
Attributes :
- Time Stamp :Wed Dec 23 18:26:31 2020

- File ID : {4E633C76-CEAE-4aa8-A518-7124A8CDF20B}
- Is CFR Compliant : false
- Original data has not been modified.

Remarks:
- User: CD
- Date: 2020/12/23
- Instrument: 0547
- DetectorType: LAAPD
- DichOS Calibration Correction Curve: 0547/2
- HV (CDDC channel): 0 v
- Time per point: 1 s
- Description: Sample 1
- Concentration: 0.0840mg/mL MeOH
- Pathlength: 1 mm
- Temperature: 20℃

Settings:
- Time-per-point: 1s (25us x 40000)
- SE
- Wavelength: 195nm - 400nm
- Step Size: 1nm
- Bandwidth: 1nm

Figure S1-7. CD and UV spectrum of (–)-**1** in MeOH

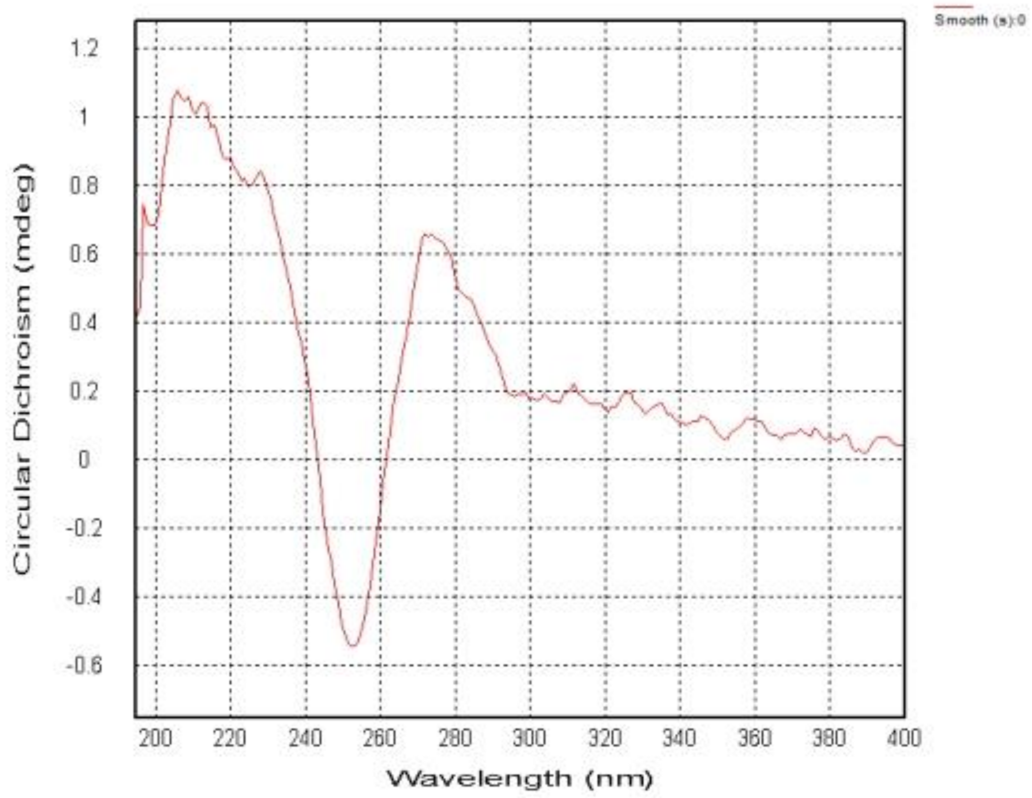

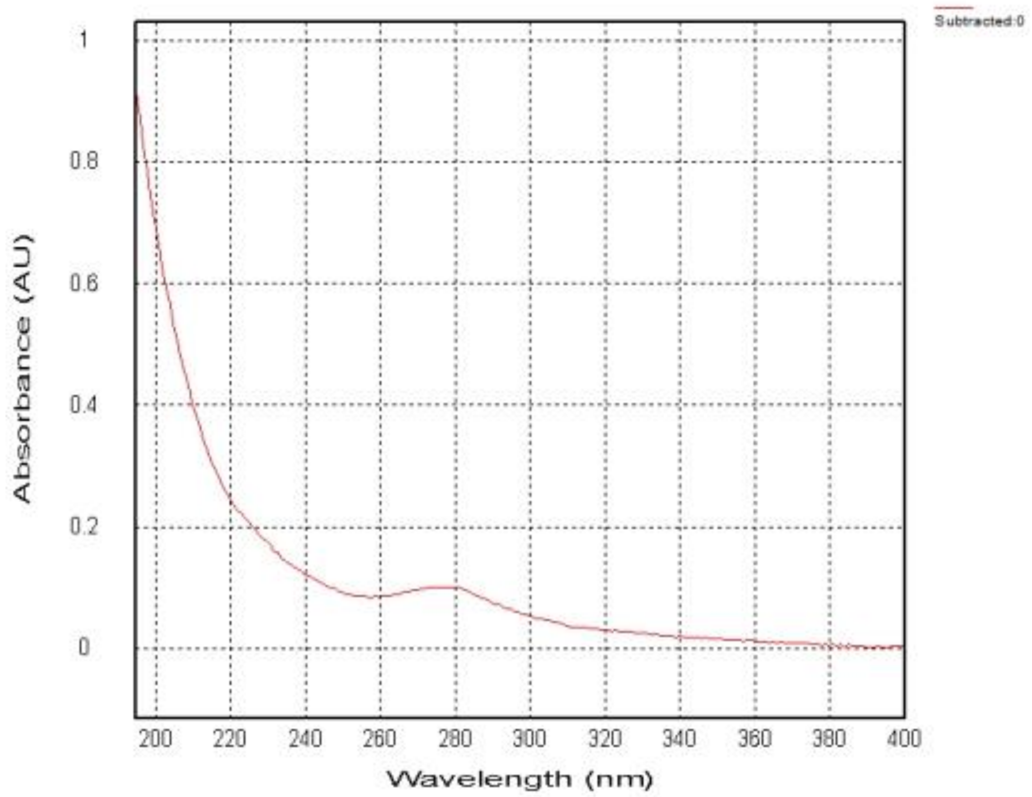

File: KMCH-13-2-1mm (195-400 nm) 20122515.dsx

ProBinaryX
Attributes :
- Time Stamp :Fri Dec 25 15:05:27 2020

- File ID : {1DADB795-454C-4c46-89C4-D53819D1C919}
- Is CFR Compliant : false
- Original data has not been modified.

Remarks:
- User: CD
- Date: 2020/12/25
- Instrument: 0547
- DetectorType: LAAPD
- DichOS Calibration Correction Curve: 0547/2
- HV (CDDC channel): 0 v
- Time per point: 1 s
- Description: Sample 1
- Concentration: 0.2100mg/mL MeOH
- Pathlength: 1 mm
- Temperature: 20℃

Settings:
- Time-per-point: 1s (25us x 40000)
- SE
- Wavelength: 195nm - 400nm
- Step Size: 1nm
- Bandwidth: 1nm

Figure S1-8. ^{1}H NMR spectrum of **1** in $CDCl_3$ (600 MHz)

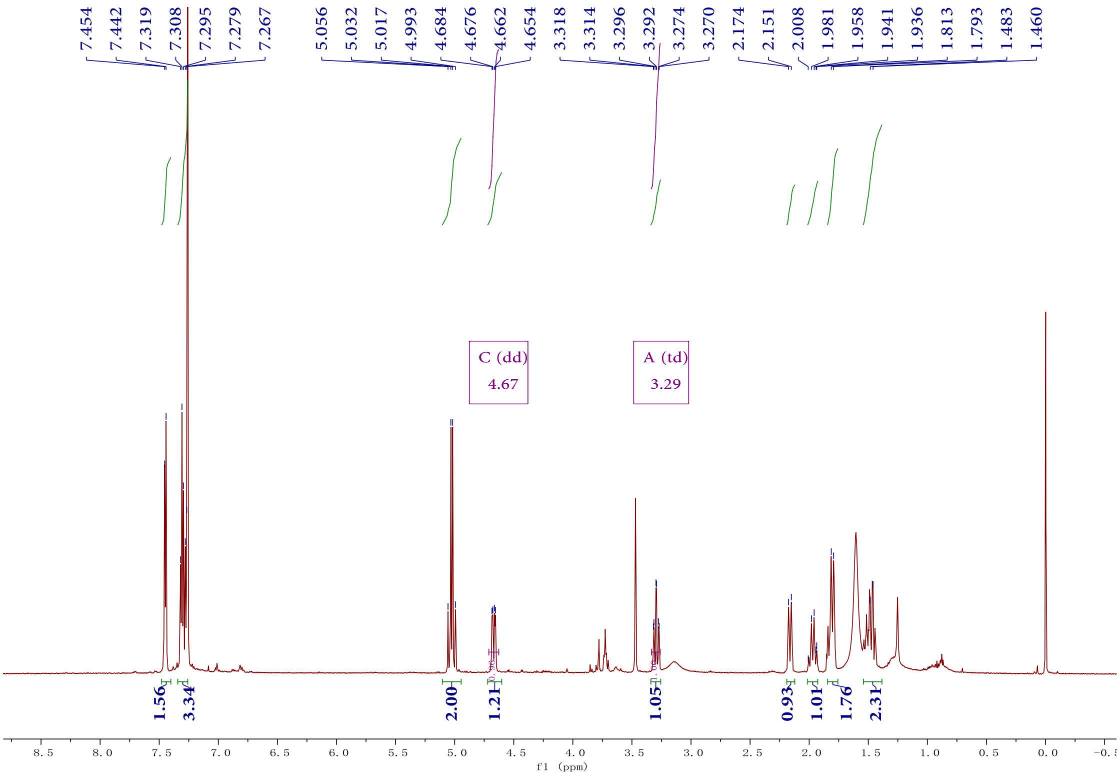

Figure S1-9. ^{13}C NMR spectrum of **1** in $CDCl_3$ (150 MHz)

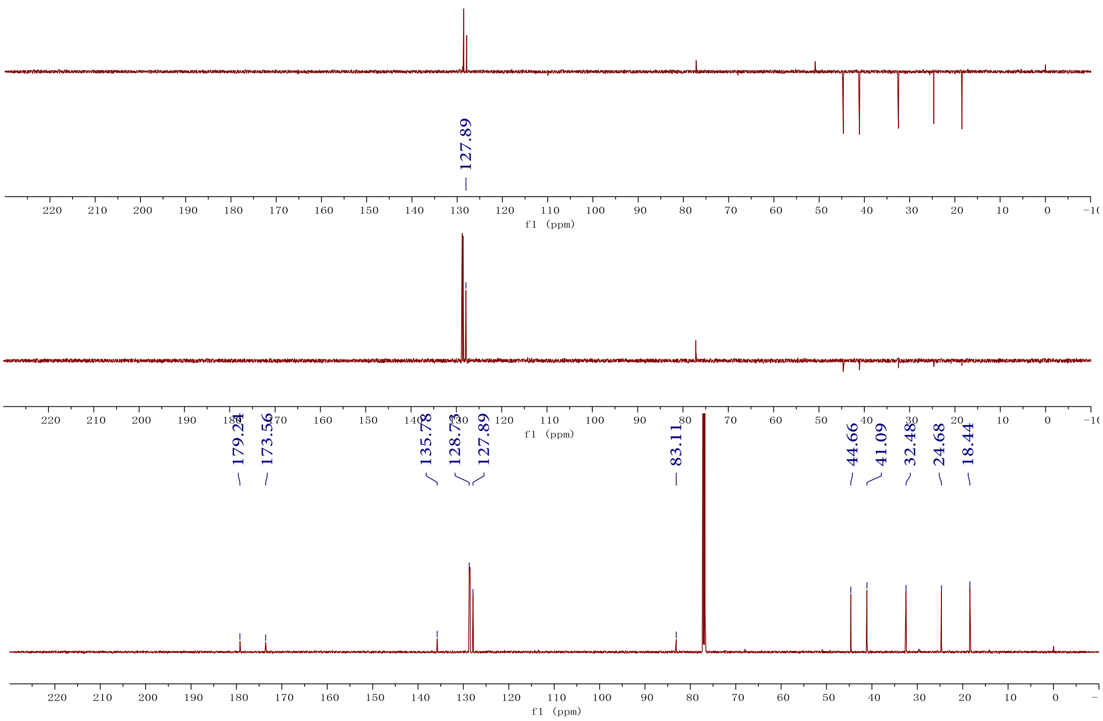

Figure S1-10. ^{1}H-^{1}H COSY spectrum of **1** in $CDCl_3$ (600 MHz)

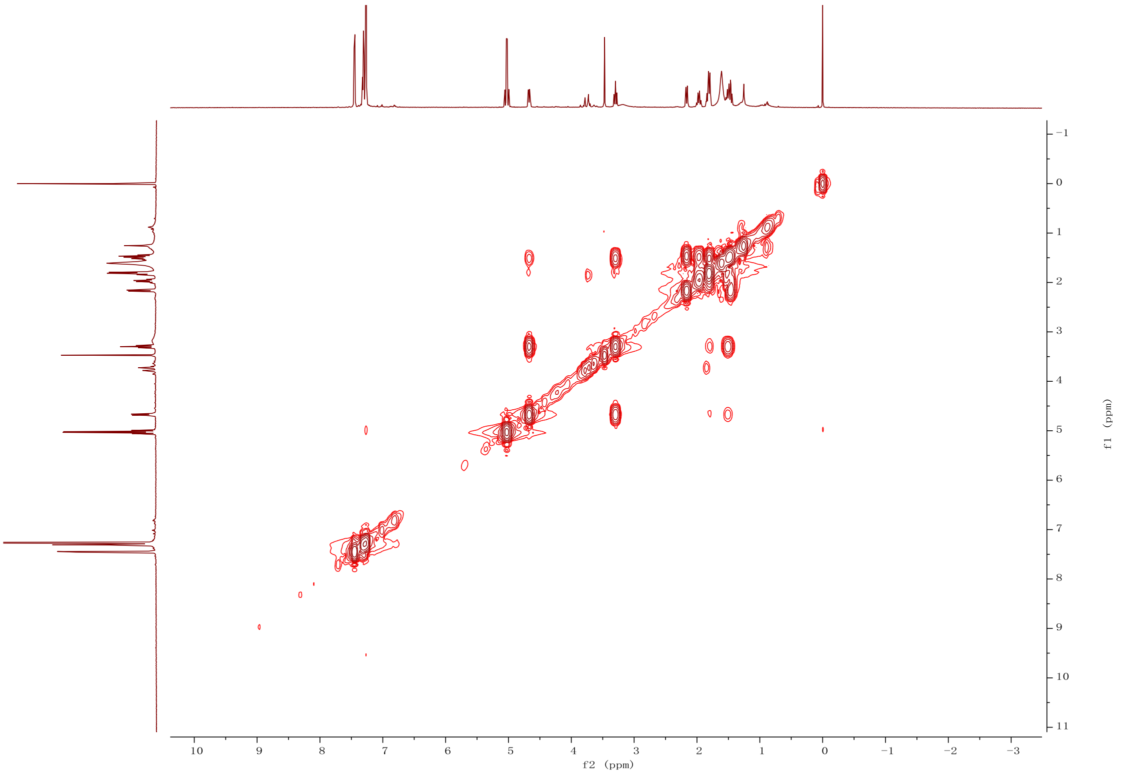

Figure S1-11. HSQC spectrum of **1** in $CDCl_3$ (600 MHz)

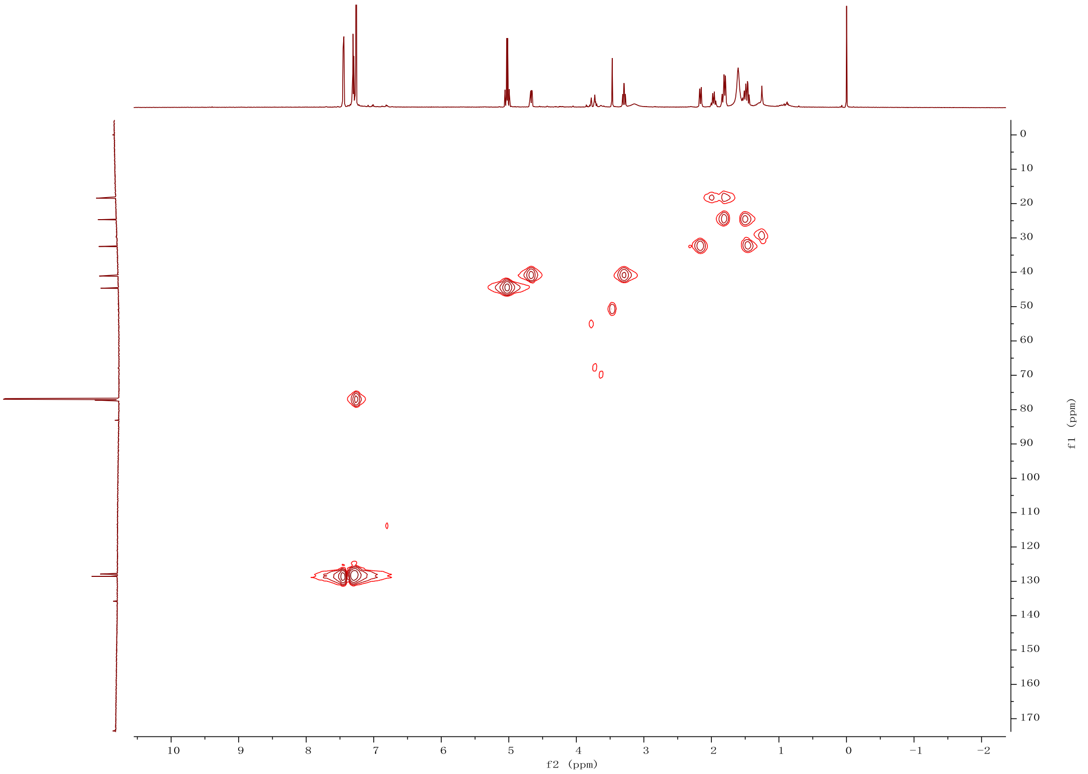

Figure S1-12 HMBC spectrum of **1** in $CDCl_3$ (600 MHz)

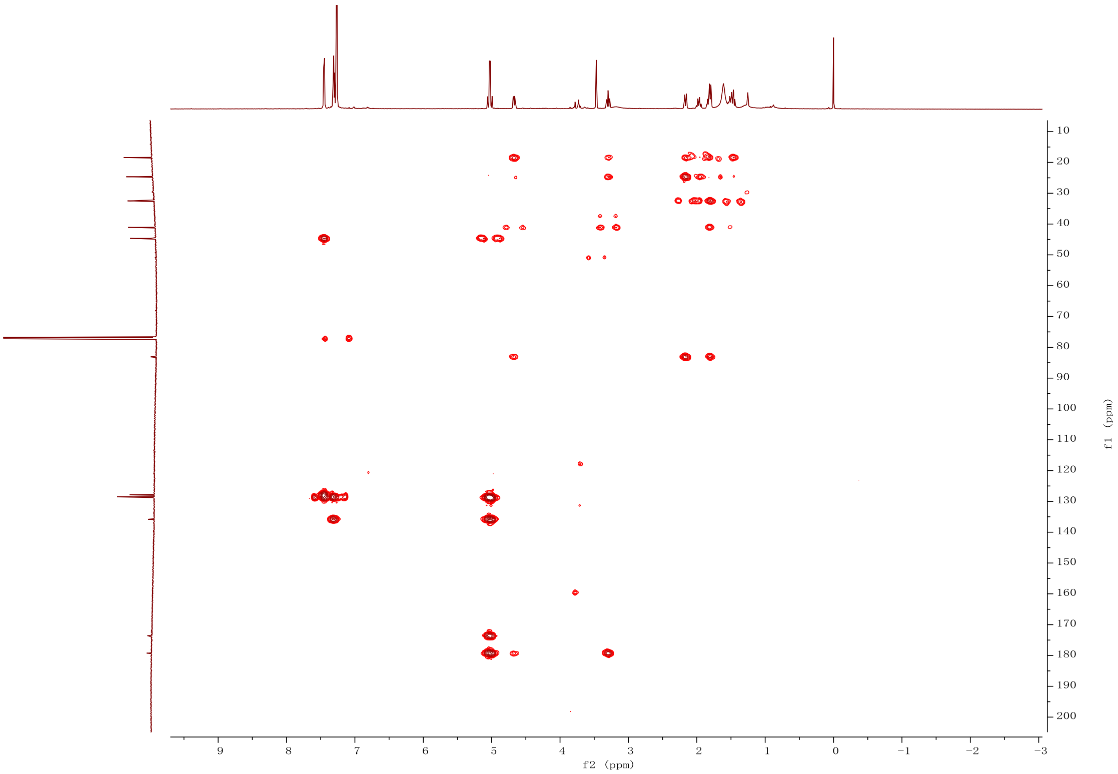

Figure S2-1. HR-ESI-MS spectrum of **2**

Qualitative Analysis Report

Data Filename	kmcr-15.d	Sample Name	kmcr-15
Sample Type	Sample	Position	P1-A5
Instrument Name	Instrument 1	User Name	
Acq Method	s.m	Acquired Time	9/16/2020 9:56:02 AM
IRM Calibration Status	Success	DA Method	Default.m
Comment			

Sample Group — Info.

Acquisition SW Version: 6200 series TOF/6500 series Q-TOF B.05.01 (B5125.2)

User Spectra

Fragmentor Voltage	Collision Energy	Ionization Mode
250	0	ESI

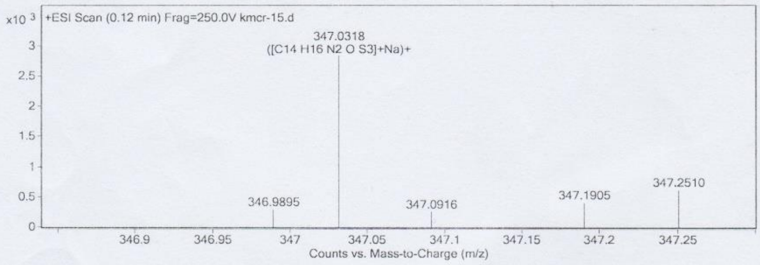

Peak List

m/z	z	Abund
268.0636	1	30148.86
298.0745	1	12434.72
301.1399	1	15696.71
413.2655	1	33746.27
414.2681	1	9448.03
429.3187	1	8311.25
591.0989	1	8331.59
701.4933	1	15617.7
814.5766	1	15494.6
815.578	1	7781.16

Formula Calculator Element Limits

Element	Min	Max
C	3	60
H	0	120
O	0	30
N	0	30
S	0	5

Formula Calculator Results

Formula	CalculatedMass	CalculatedMz	Mz	Diff. (mDa)	Diff. (ppm)	DBE
C14 H16 N2 O S3	324.0425	347.0317	347.0318	-0.10	-0.29	8.0000

--- End Of Report ---

Agilent Technologies — Page 1 of 1 — Printed at: 11:12 AM on: 9/17/2020

Figure S2-2 ESI-MS spectrum of **2**

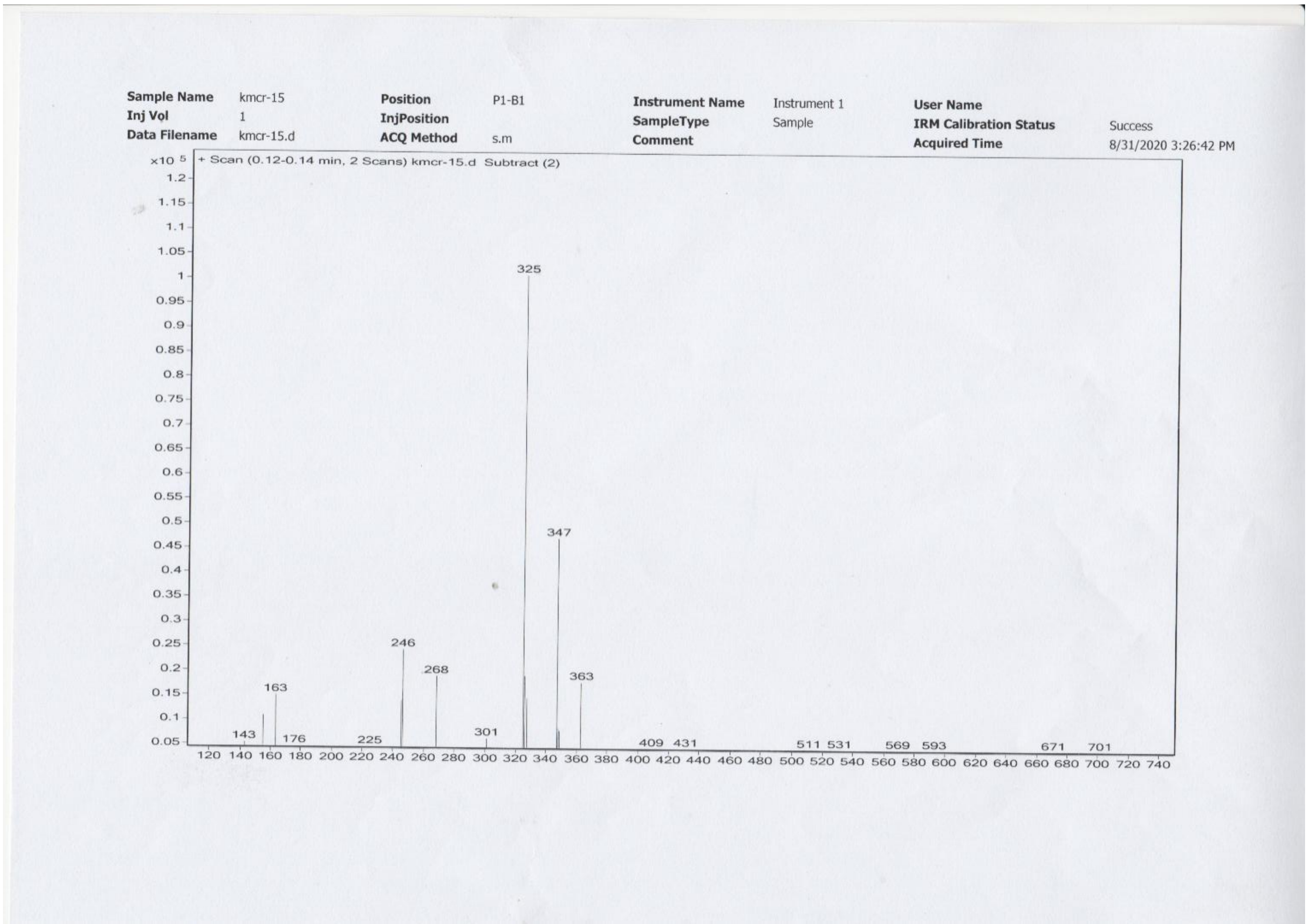

Figure S2-3 The chiral analytical HPLC chromatography of **2**

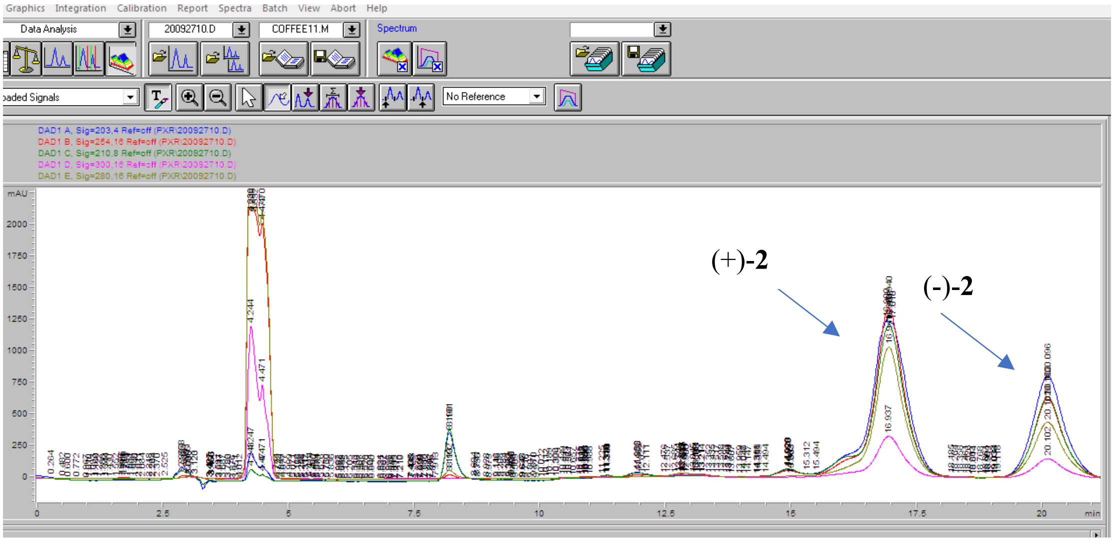

Figure S2-4. $[\alpha]_D$ data of (+)-**2**

Rudolph Research Analytical

This sample was measured on an Autopol VI, Serial #91058
Manufactured by Rudolph Research Analytical, Hackettstown, NJ, USA.

Measurement Date : Monday, 12-OCT-2020

Set Temperature : OFF

Time Delay : Disabled

Delay between Measurement : Disabled

n	Average	Std.Dev.	% RSD	Maximum	Minimum
5	24.04	0.19	0.79	24.32	23.79

S.No	Sample ID	Time	Result	Scale	OR °Arc	WLG.nm	Lg.mm	Conc.g/100ml	Temp.
1	KMCR-15-1	02:28:09 PM	24.05	SR	0.0457	589	100.00	0.190	24.9
2	KMCR-15-1	02:28:18 PM	24.05	SR	0.0457	589	100.00	0.190	24.9
3	KMCR-15-1	02:28:26 PM	24.32	SR	0.0462	589	100.00	0.190	24.9
4	KMCR-15-1	02:28:34 PM	24.00	SR	0.0456	589	100.00	0.190	24.9
5	KMCR-15-1	02:28:42 PM	23.79	SR	0.0452	589	100.00	0.190	24.9

Figure S2-5. $[\alpha]_D$ data of (–)-**2**

Rudolph Research Analytical

This sample was measured on an Autopol VI, Serial #91058
Manufactured by Rudolph Research Analytical, Hackettstown, NJ, USA.

Measurement Date : Monday, 12-OCT-2020

Set Temperature : OFF

Time Delay : Disabled

Delay between Measurement : Disabled

n	Average	Std.Dev.	% RSD	Maximum	Minimum
5	-10.63	0.15	-1.41	-10.44	-10.81

S.No	Sample ID	Time	Result	Scale	OR °Arc	WLG.nm	Lg.mm	Conc.g/100ml	Temp.
1	KMCR-15-2	02:42:56 PM	-10.81	SR	-0.0173	589	100.00	0.160	24.7
2	KMCR-15-2	02:43:04 PM	-10.69	SR	-0.0171	589	100.00	0.160	24.7
3	KMCR-15-2	02:43:12 PM	-10.44	SR	-0.0167	589	100.00	0.160	24.8
4	KMCR-15-2	02:43:21 PM	-10.69	SR	-0.0171	589	100.00	0.160	24.8
5	KMCR-15-2	02:43:29 PM	-10.50	SR	-0.0168	589	100.00	0.160	24.8

Figure S2-6. CD and UV spectrum of (+)-**2** in MeOH

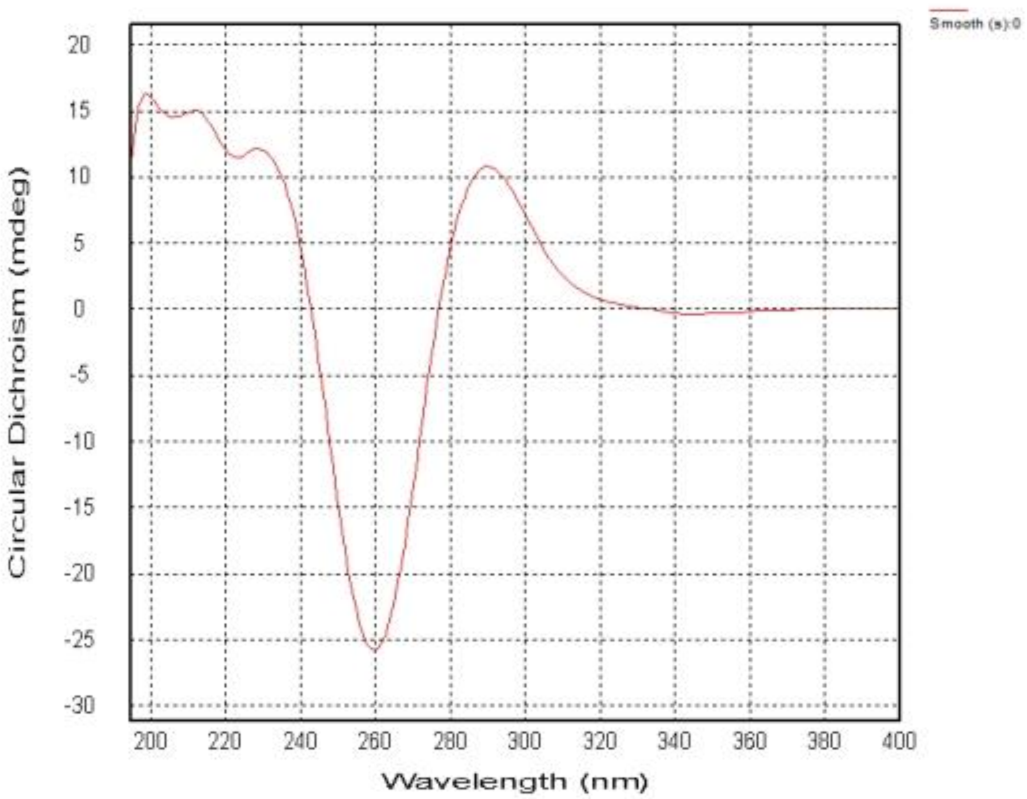

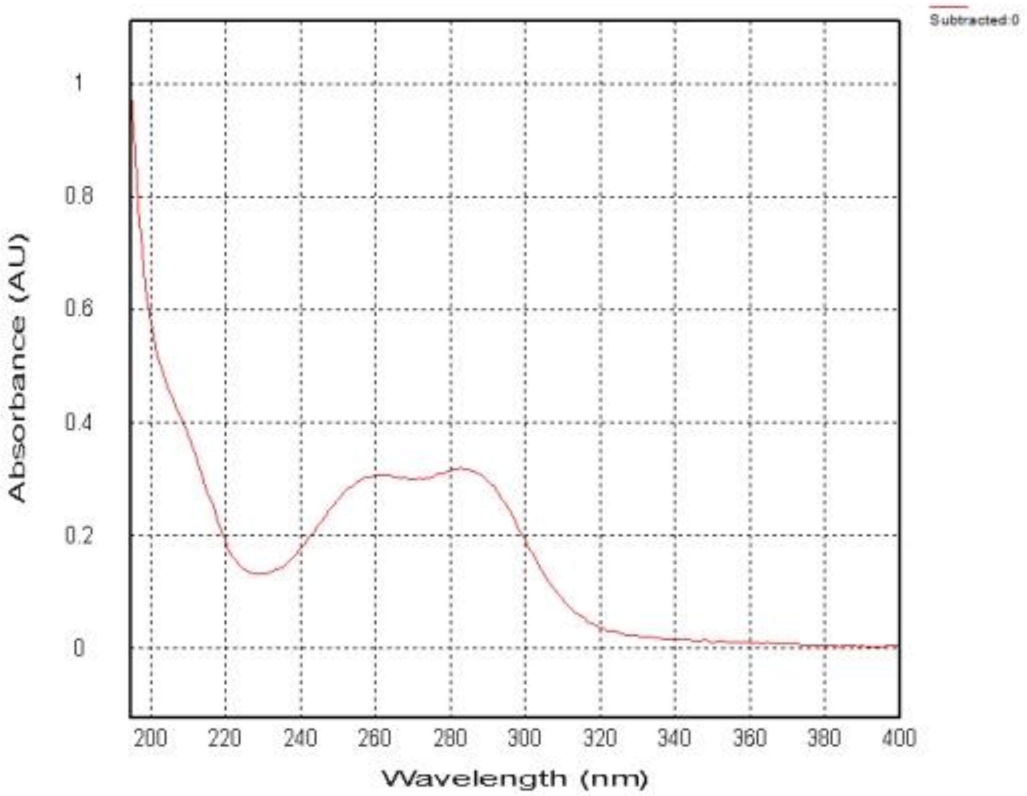

File: KMCR-15-1-1mm (195-400 nm) 20101317.dsx
ProBinaryX
Attributes :
- Time Stamp :Tue Oct 13 14:17:23 2020

- File ID : {0D1C595B-753D-4a0d-B783-C3CD5203F4AA}
- Is CFR Compliant : false
- Original data has not been modified.

Remarks:
- User: CD
- Date: 2020/10/13
- Instrument: 0547
- DetectorType: LAAPD
- DichOS Calibration Correction Curve: 0547/2
- HV (CDDC channel): 0 v
- Time per point: 1 s
- Description: Sample 1
- Concentration: 0.0821mg/mL MeOH
- Pathlength: 1 mm
- Temperature: 20℃

Settings:
- Time-per-point: 1s (25us x 40000)
- SE
- Wavelength: 195nm - 400nm
- Step Size: 1nm
- Bandwidth: 1nm

Figure S2-7. CD and UV spectrum of (–)-**2** in MeOH

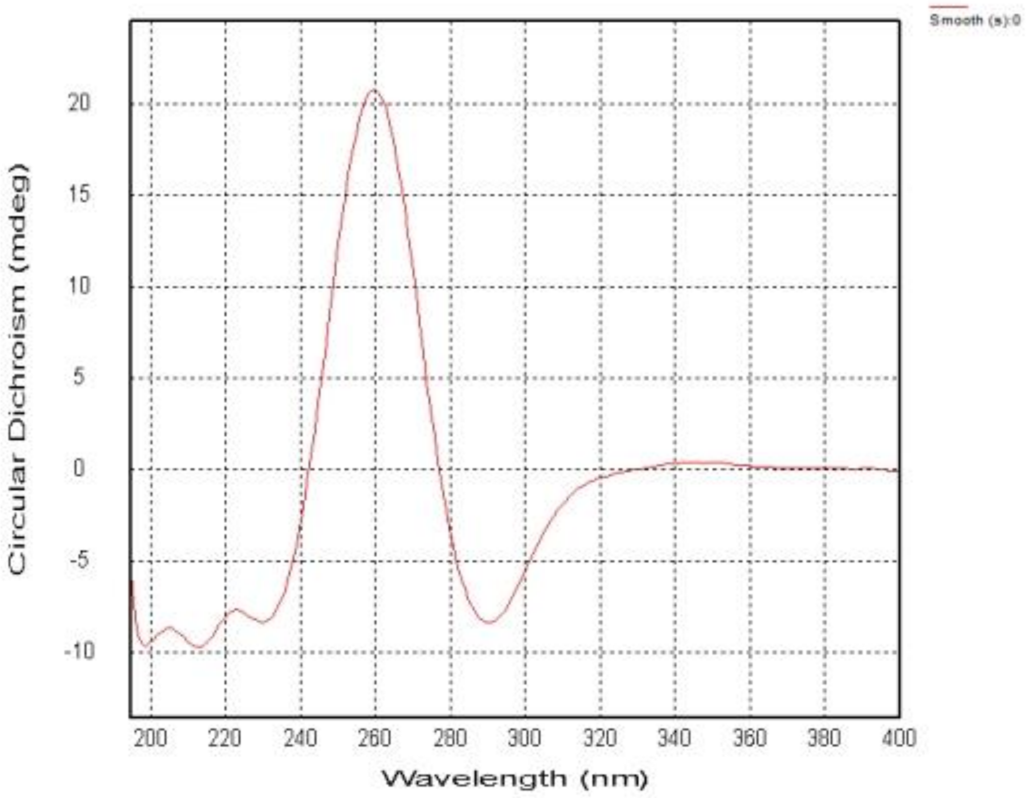

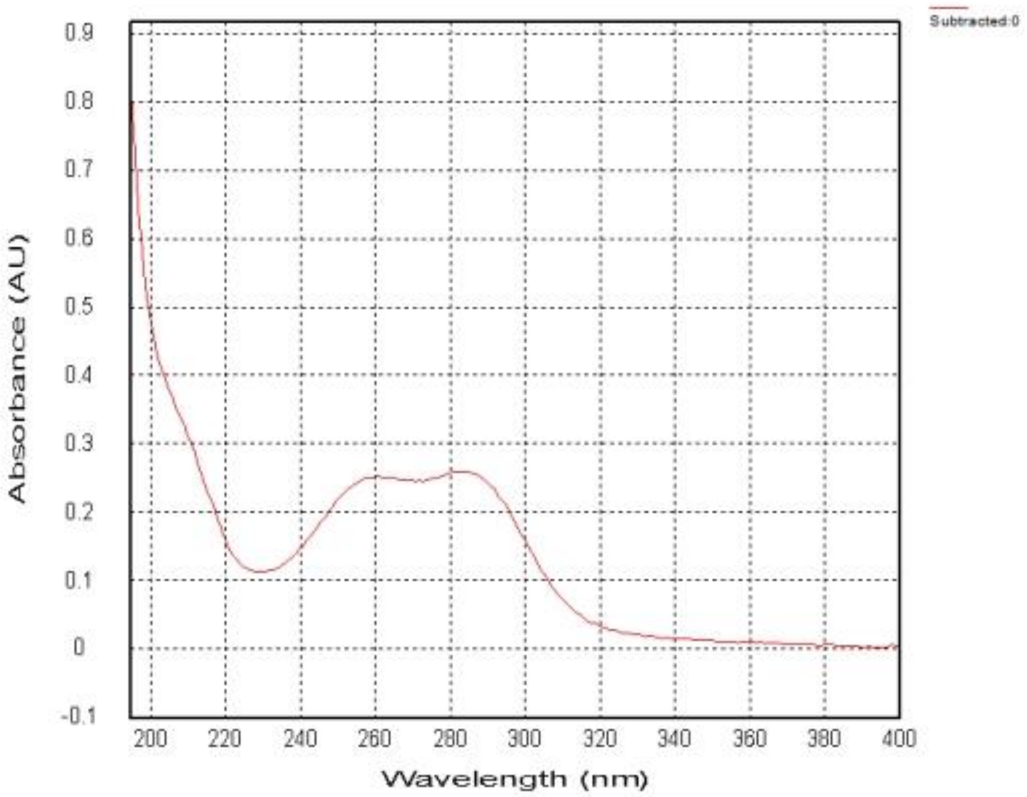

File: KMCR-15-2-1mm (195-400 nm) 20101318.dsx

ProBinaryX
Attributes :
- Time Stamp :Tue Oct 13 14:34:07 2020

- File ID : {DC13B8B9-40B0-43c7-951A-22B86084DB5F}
- Is CFR Compliant : false
- Original data has not been modified.
Remarks:
- User: CD
- Date: 2020/10/13
- Instrument: 0547
- DetectorType: LAAPD
- DichOS Calibration Correction Curve: 0547/2
- HV (CDDC channel): 0 v
- Time per point: 1 s
- Description: Sample 1
- Concentration: 0.0576mg/mL MeOH
- Pathlength: 1 mm
- Temperature: 20℃

Settings:
- Time-per-point: 1s (25us x 40000)
- SE
- Wavelength: 195nm - 400nm
- Step Size: 1nm
- Bandwidth: 1nm

Figure S2-8. ^{1}H NMR spectrum of **2** in $CDCl_3$ (600 MHz)

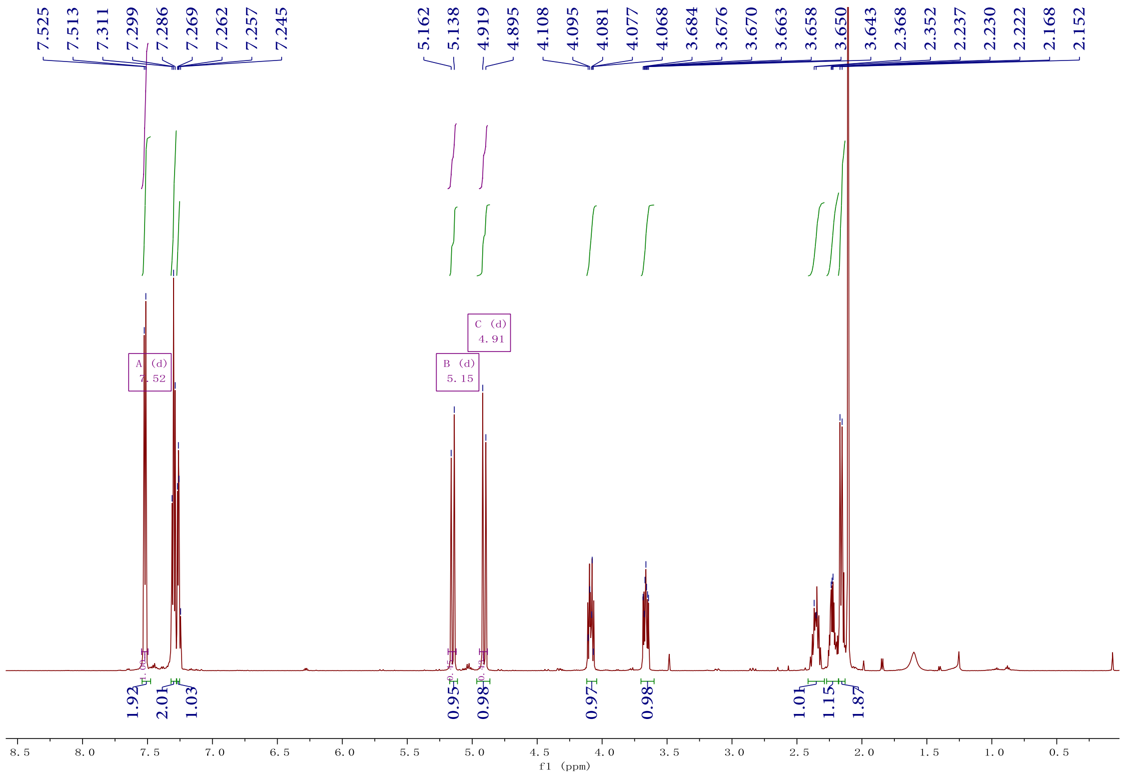

Figure S2-9. ^{13}C NMR spectrum of **2** in $CDCl_3$ (150 MHz)

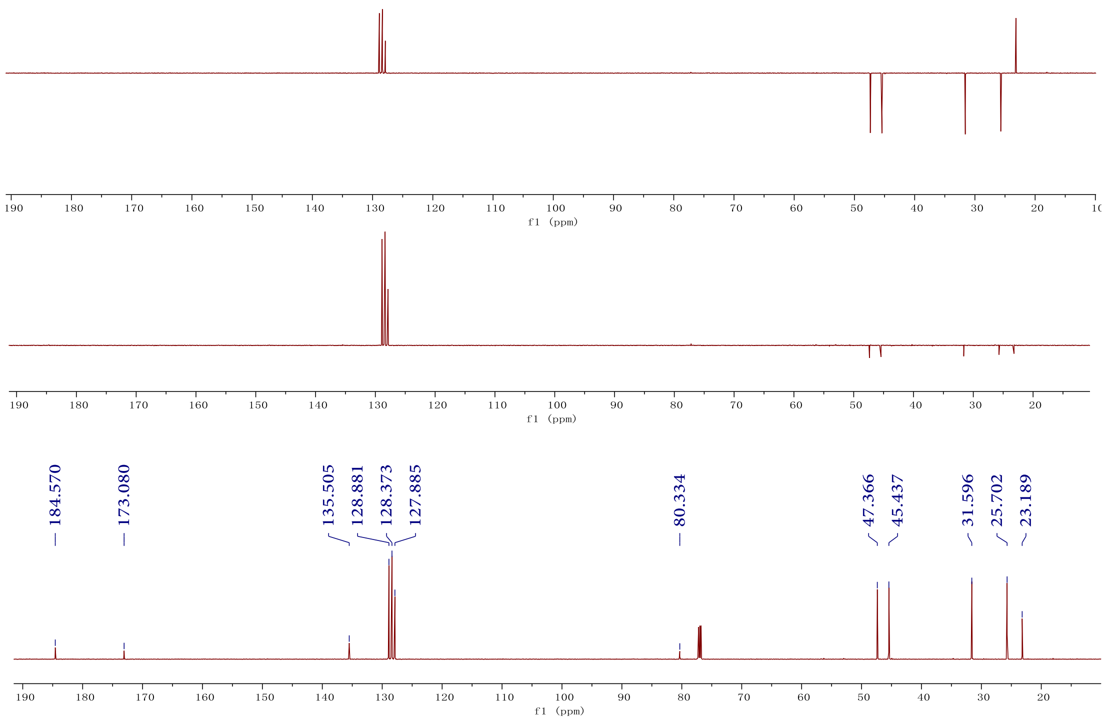

Figure S2-10. ^1H-^1H COSY spectrum of **2** in $CDCl_3$ (600 MHz)

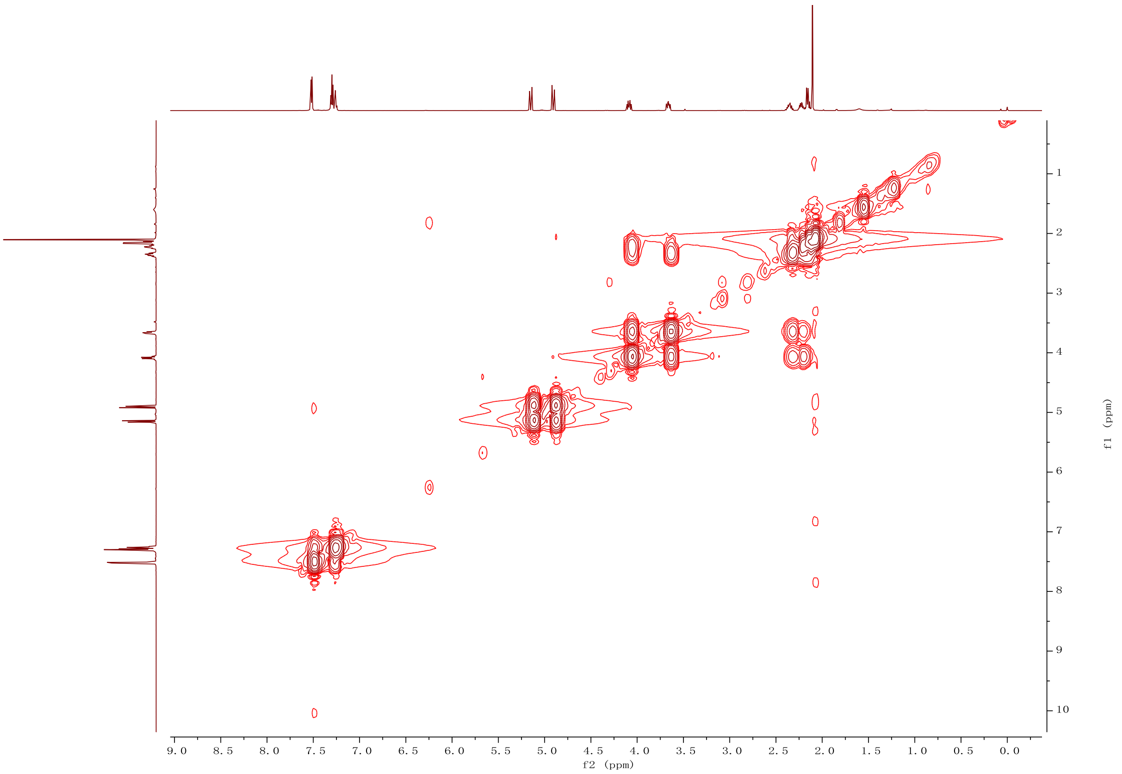

Figure S2-11. HSQC spectrum of **2** in $CDCl_3$ (600 MHz)

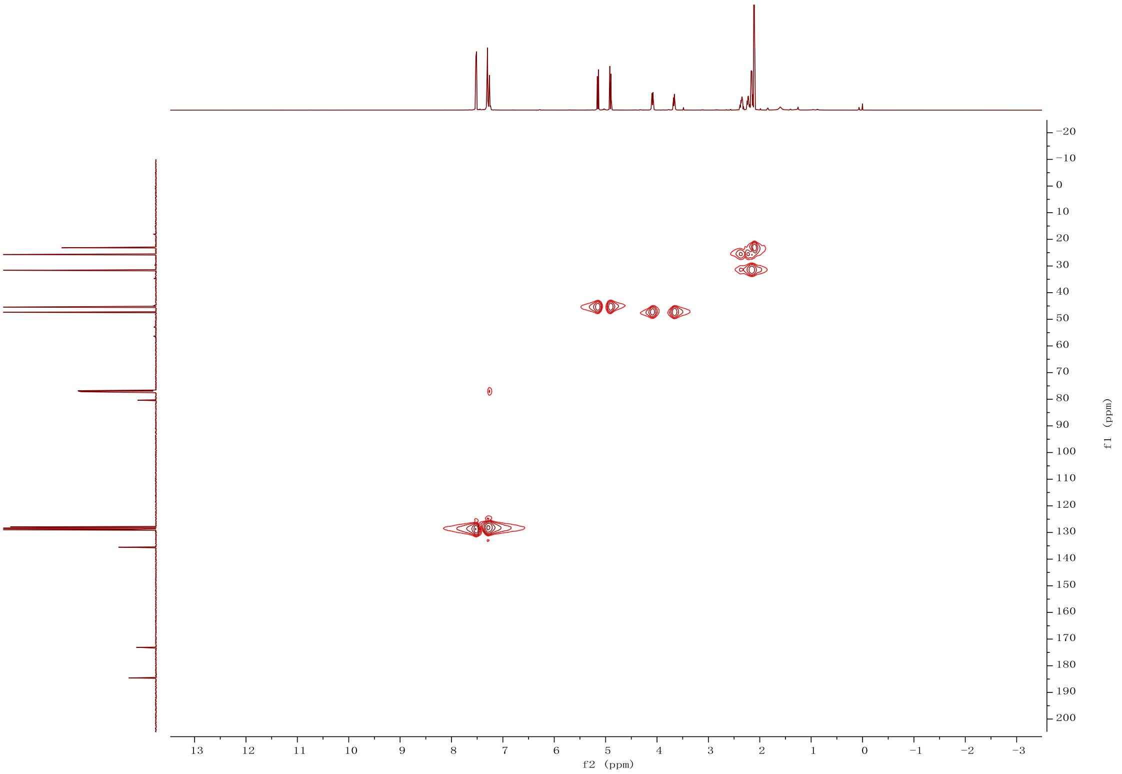

Figure S2-12 HMBC spectrum of **2** in $CDCl_3$ (600 MHz)

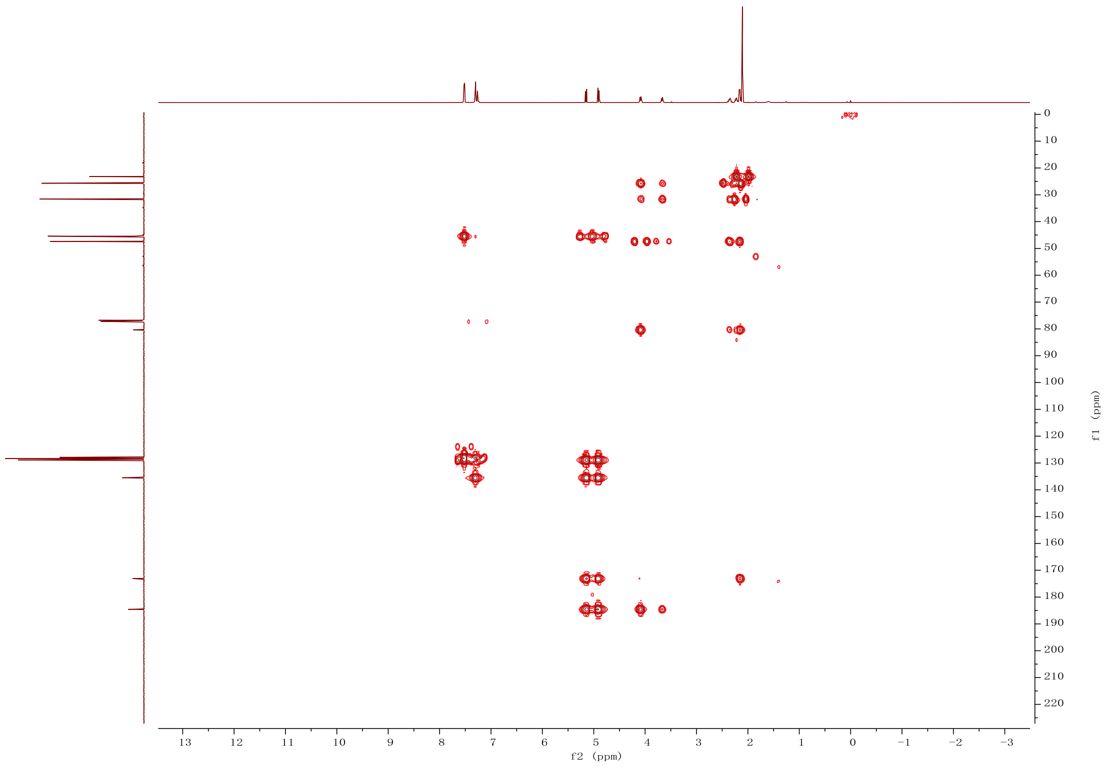

Figure S3-1. HR-ESI-MS spectrum of **3**

Qualitative Analysis Report

Data Filename	kmcr-10.d	Sample Name	kmcr-10
Sample Type	Sample	Position	P1-A1
Instrument Name	Instrument 1	User Name	
Acq Method	s.m	Acquired Time	8/7/2020 3:57:46 PM
IRM Calibration Status	Success	DA Method	Default.m
Comment			

Sample Group		Info.
Acquisition SW Version	6200 series TOF/6500 series Q-TOF B.05.01 (B5125.2)	

User Spectra

Fragmentor Voltage	Collision Energy	Ionization Mode
80	0	ESI

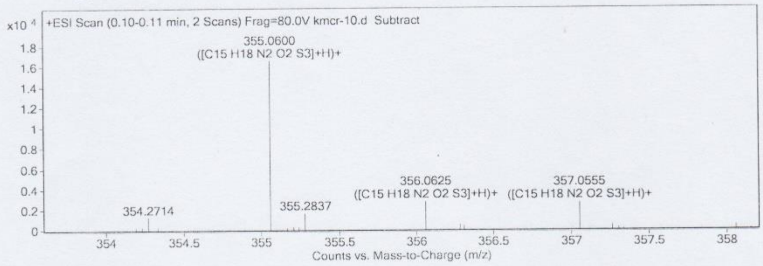

Peak List

m/z	z	Abund	Formula	Ion
102.1278	1	16062.6		
150.1124		4200.75		
279.1581	1	4396.01		
323.2575	1	4928.07		
325.2724	1	7138.06		
332.294	1	6751.22		
355.06	1	16547.01	C15 H18 N2 O2 S3	(M+H)+
363.2492	1	6869.92		
365.2654	1	11512.51		
393.2603	1	7588.96		

Formula Calculator Element Limits

Element	Min	Max
C	3	60
H	0	120
O	0	30
N	0	5
S	0	5
Cl	0	5

Formula Calculator Results

Formula	CalculatedMass	CalculatedMz	Mz	Diff. (mDa)	Diff. (ppm)	DBE
C15 H18 N2 O2 S3	354.0530	355.0603	355.0600	0.30	0.84	8.0000

--- End Of Report ---

Agilent Technologies Page 1 of 1 Printed at: 3:42 PM on: 9/10/2020

Figure S3-2. ESI-MS spectrum of **3**

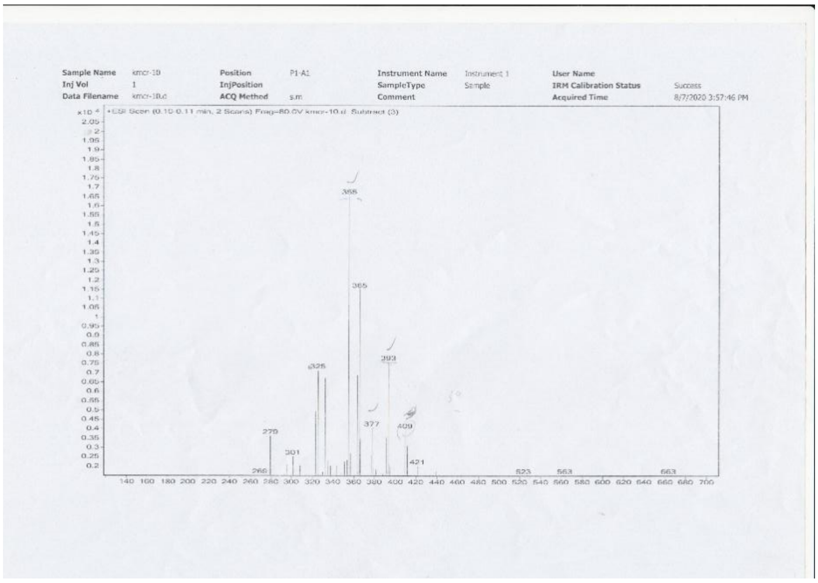

Figure S3-2 The chiral analytical HPLC chromatography of **3**

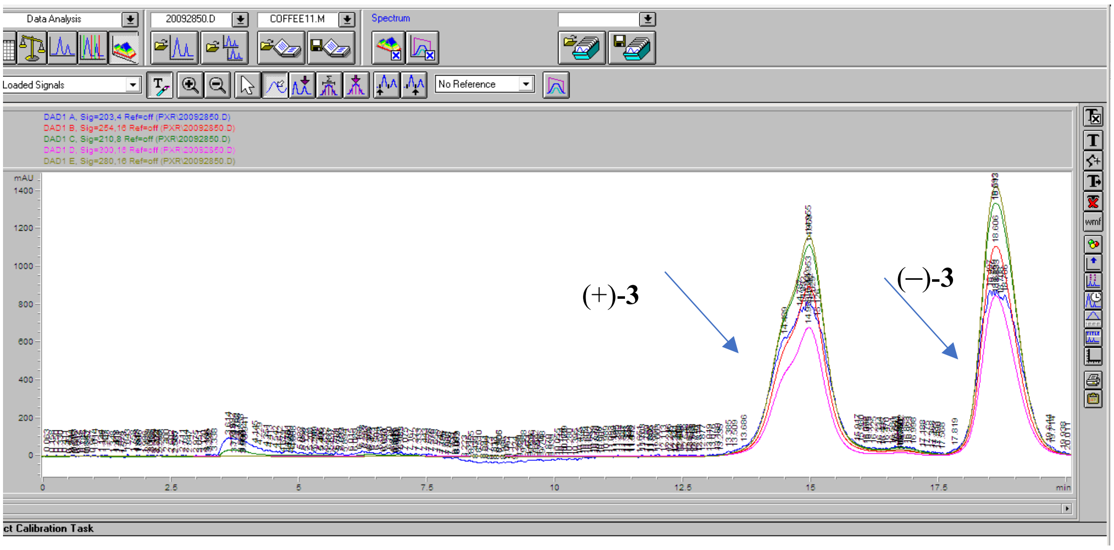

Figure S3-3. [α] D data of (+)-**3**

Rudolph Research Analytical

This sample was measured on an Autopol VI, Serial #91058
Manufactured by Rudolph Research Analytical, Hackettstown, NJ, USA.

Measurement Date : Monday, 12-OCT-2020

Set Temperature : OFF

Time Delay : Disabled

Delay between Measurement : Disabled

n	Average	Std.Dev.	% RSD	Maximum	Minimum
5	37.38	0.31	0.82	37.63	36.88

S.No	Sample ID	Time	Result	Scale	OR °Arc	WLG.nm	Lg.mm	Conc.g/100ml	Temp.
1	KMCR-10-1	01:55:12 PM	37.63	SR	0.0301	589	100.00	0.080	24.3
2	KMCR-10-1	01:55:20 PM	36.88	SR	0.0295	589	100.00	0.080	24.3
3	KMCR-10-1	01:55:28 PM	37.38	SR	0.0299	589	100.00	0.080	24.4
4	KMCR-10-1	01:55:36 PM	37.38	SR	0.0299	589	100.00	0.080	24.4
5	KMCR-10-1	01:55:44 PM	37.63	SR	0.0301	589	100.00	0.080	24.4

Figure S3-4. $[\alpha]_D$ data of (–)-**3**

Rudolph Research Analytical

This sample was measured on an Autopol VI, Serial #91058
Manufactured by Rudolph Research Analytical, Hackettstown, NJ, USA.

Measurement Date : Monday, 12-OCT-2020

Set Temperature : OFF

Time Delay : Disabled

Delay between Measurement : Disabled

n	Average	Std.Dev.	% RSD	Maximum	Minimum
5	-38.44	0.33	-0.85	-38.20	-39.00

S.No	Sample ID	Time	Result	Scale	OR °Arc	WLG.nm	Lg.mm	Conc.g/100ml	Temp.
1	KMCR-10-2	02:16:05 PM	-38.20	SR	-0.0191	589	100.00	0.050	24.9
2	KMCR-10-2	02:16:13 PM	-39.00	SR	-0.0195	589	100.00	0.050	24.9
3	KMCR-10-2	02:16:21 PM	-38.40	SR	-0.0192	589	100.00	0.050	24.9
4	KMCR-10-2	02:16:29 PM	-38.20	SR	-0.0191	589	100.00	0.050	24.9
5	KMCR-10-2	02:16:37 PM	-38.40	SR	-0.0192	589	100.00	0.050	24.9

Figure S3-5. CD and UV spectrum of (+)-**3** in MeOH

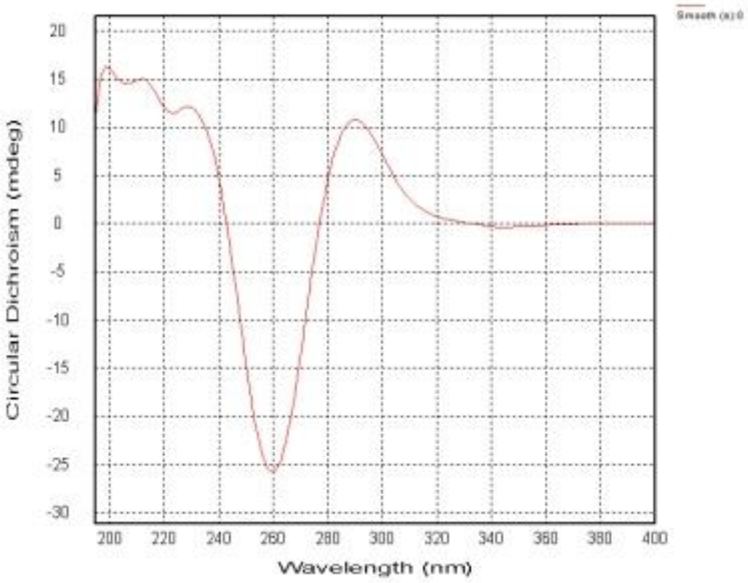

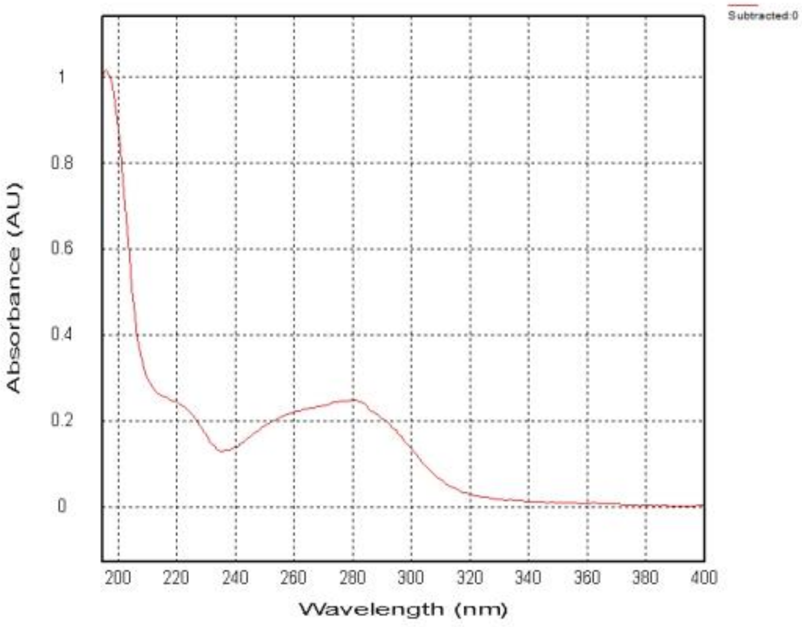

File: KMCR-10-1-1mm (195-400 nm) 20101314.dsx
ProBinaryX
Attributes :
- Time Stamp :Tue Oct 13 13:23:11 2020

- File ID : {7F932724-A7E0-4c10-9846-AD5C6E93BD92}
- Is CFR Compliant : false
- Original data has not been modified.
Remarks:
 - User: CD
 - Date: 2020/10/13
 - Instrument: 0547
 - DetectorType: LAAPD
 - DichOS Calibration Correction Curve: 0547/2
 - HV (CDDC channel): 0 v
 - Time per point: 1 s
 - Description: Sample 1
 - Concentration: 0.0461mg/mL MeOH
 - Pathlength: 1 mm
 - Temperature: 20℃

Settings:
 - Time-per-point: 1s (25us x 40000)
 - SE
 - Wavelength: 195nm - 400nm
 - Step Size: 1nm
 - Bandwidth: 1nm

Figure S3-6. CD and UV spectrum of (–)-**3** in MeOH

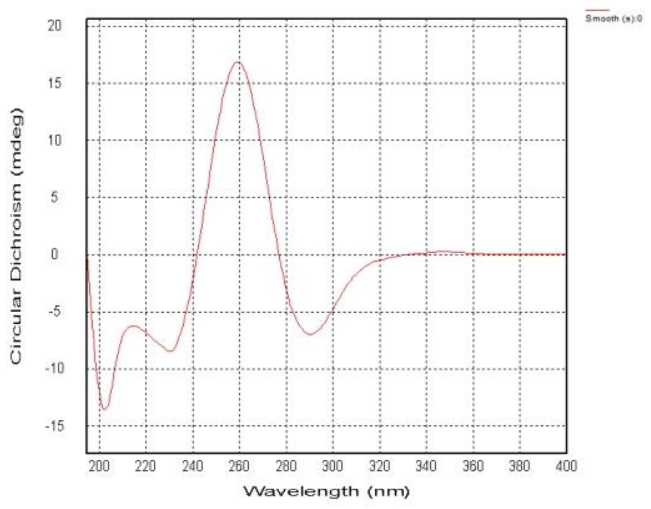

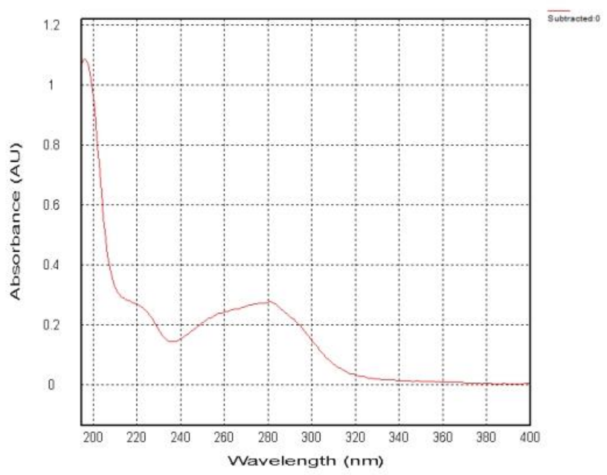

File: KMCR-10-2-1mm (195-400 nm) 20101315.dsx
ProBinaryX
Attributes :
- Time Stamp :Tue Oct 13 13:41:19 2020

- File ID : {57E8BF71-1B1F-4b0d-9BFF-8D1E35EE633B}
- Is CFR Compliant : false
- Original data has not been modified.

Remarks:
- User: CD
- Date: 2020/10/13
- Instrument: 0547
- DetectorType: LAAPD
- DichOS Calibration Correction Curve: 0547/2
- HV (CDDC channel): 0 v
- Time per point: 1 s
- Description: Sample 1
- Concentration: 0.0324mg/mL MeOH
- Pathlength: 1 mm
- Temperature: 20℃

Settings:
- Time-per-point: 1s (25us x 40000)
- SE
- Wavelength: 195nm - 400nm
- Step Size: 1nm
- Bandwidth: 1nm

Figure S3-7. ^{1}H NMR spectrum of **3** in $CDCl_3$ (600 MHz)

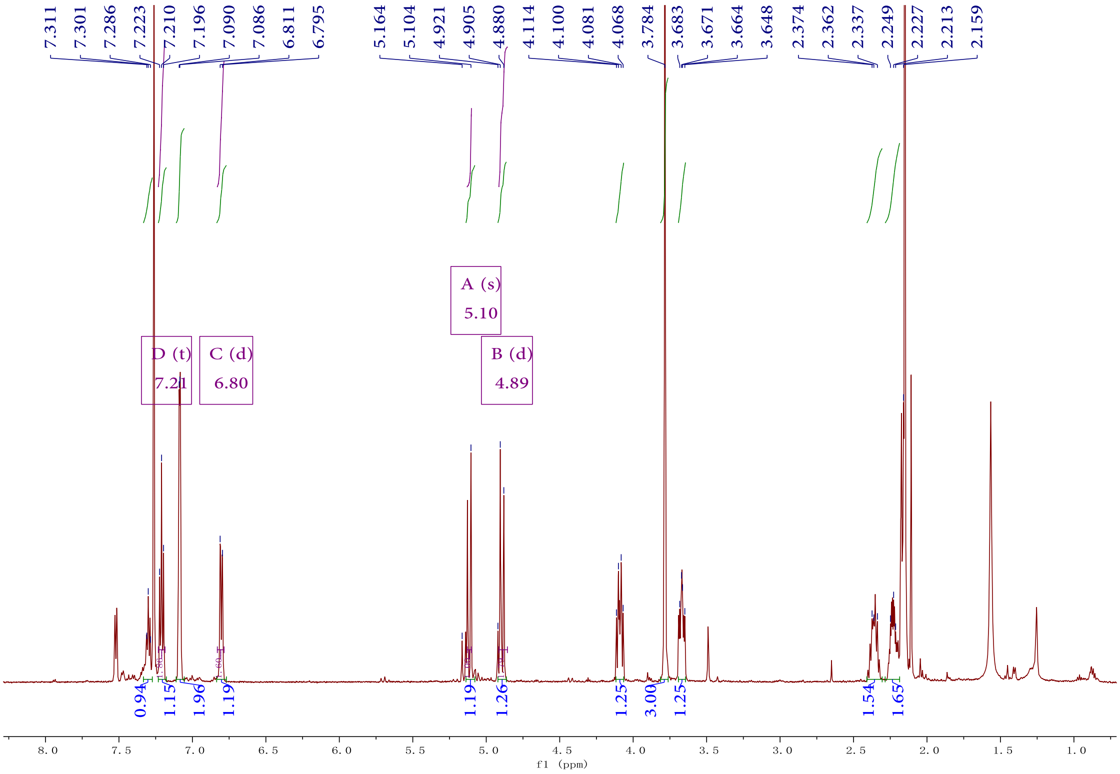

Figure S3-8. ^{13}C NMR spectrum of **3** in $CDCl_3$ (150 MHz)

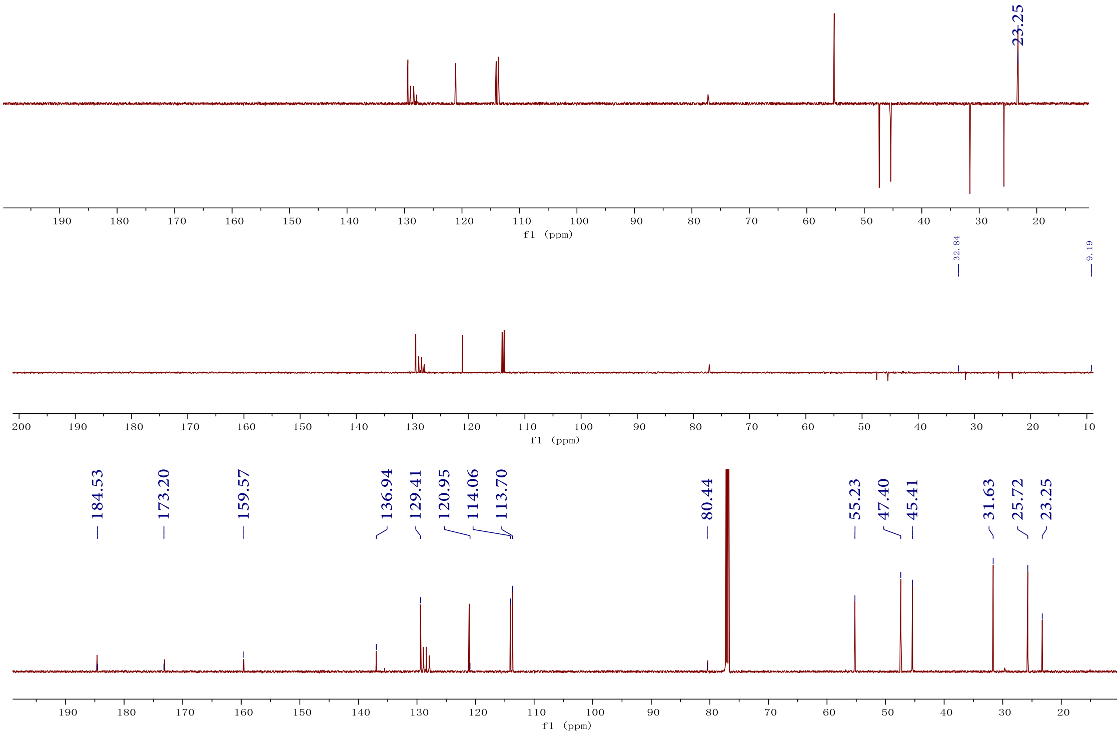

Figure S3-9. ^1H-^1H COSY spectrum of **3** in $CDCl_3$ (600 MHz)

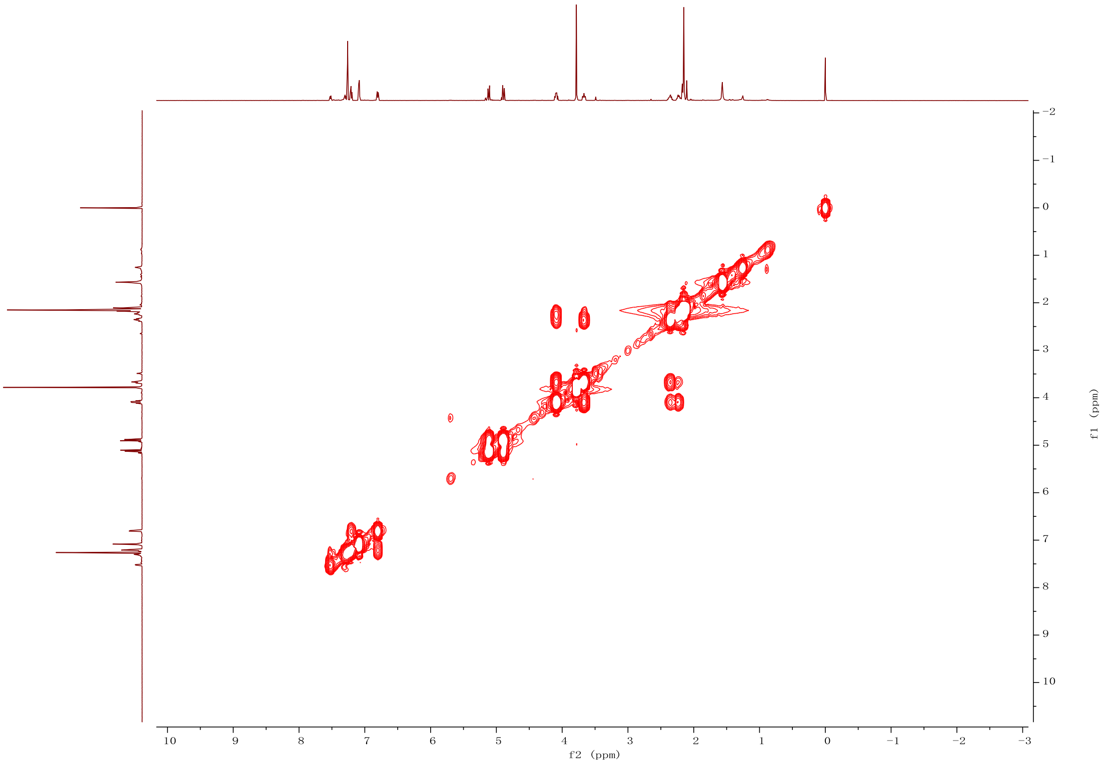

Figure S3-10. HSQC spectrum of **3** in $CDCl_3$ (600 MHz)

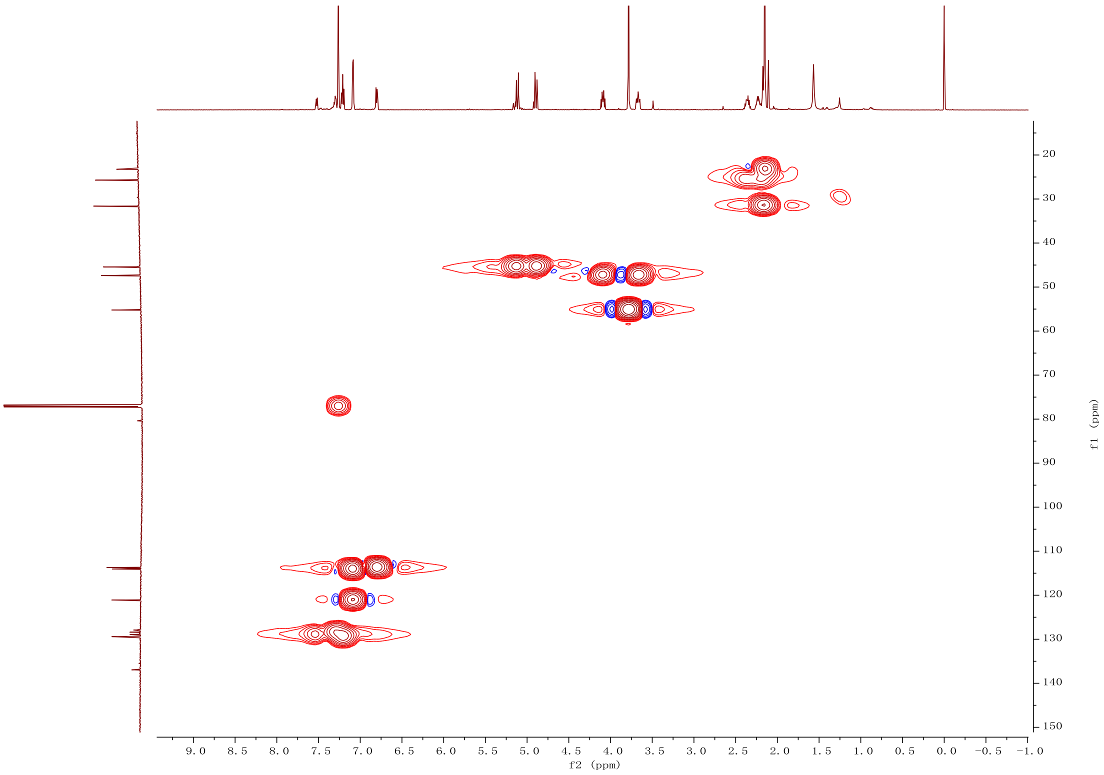

Figure S3-11 HMBC spectrum of **3** in $CDCl_3$ (600 MHz)

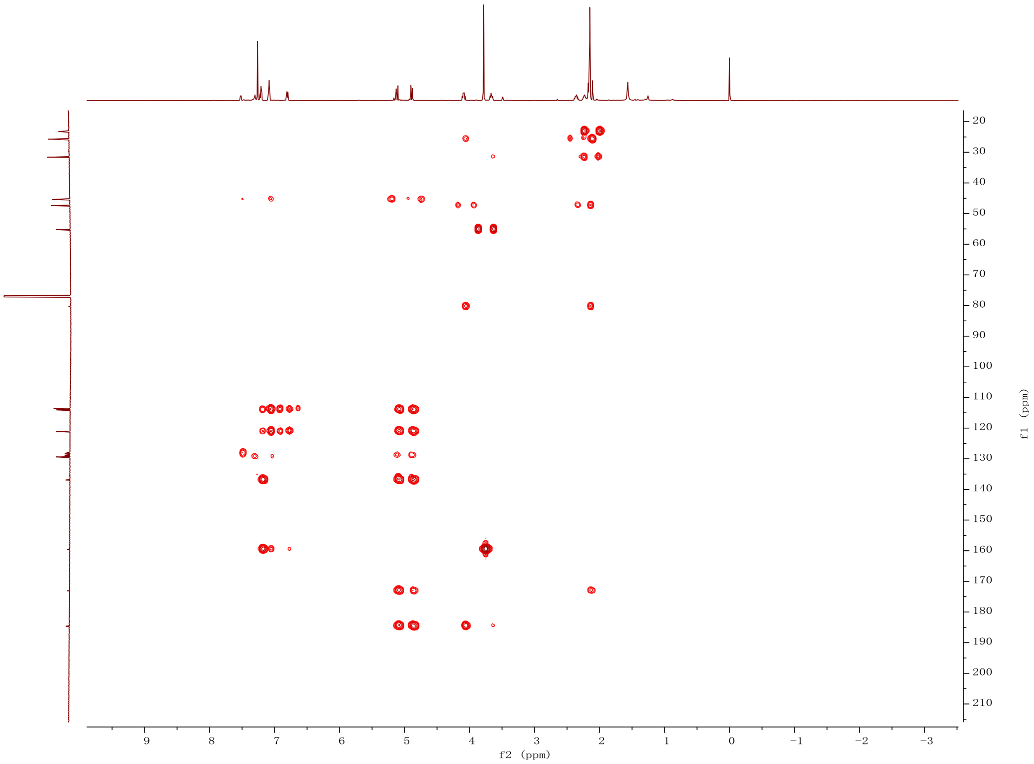

Figure S4-1. HR-ESI-MS spectrum of **4**

Qualitative Analysis Report

**Data Filename**	kzm853nn.d	**Sample Name**	kzm853nn
**Sample Type**	Sample	**Position**	P1-B8
**Instrument Name**	Instrument 1	**User Name**	
**Acq Method**	s-.m	**Acquired Time**	9/16/2020 11:22:57 AM
**IRM Calibration Status**	Success	**DA Method**	Default.m
**Comment**			

Sample Group **Info.**

Acquisition SW 6200 series TOF/6500 series
Version Q-TOF B.05.01 (B5125.2)

User Spectra

Fragmentor Voltage	Collision Energy	Ionization Mode
135	0	ESI

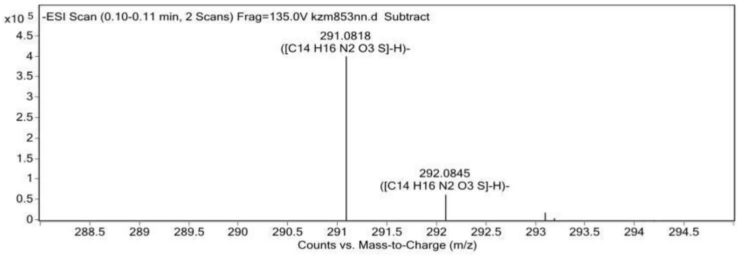

Peak List

m/z	z	Abund	Formula	Ion
185.0394		48709.22		
255.2336	1	199032.97		
261.0708	1	56235.94		
283.2651	1	111140.55		
291.0818	1	400975.81	C14 H16 N2 O3 S	(M-H)-
292.0845	1	65118.77	C14 H16 N2 O3 S	(M-H)-
311.1697	1	88956.39		
325.1853	1	159172.33		
339.2011	1	179109.22		
547.3228	1	104347.63		

Formula Calculator Element Limits

Element	Min	Max
C	3	60
H	0	120
O	0	30
N	0	30
S	0	5

Formula Calculator Results

Formula	CalculatedMass	CalculatedMz	Mz	Diff. (mDa)	Diff. (ppm)	DBE
C14 H16 N2 O3 S	292.0882	291.0809	291.0818	-0.90	-3.09	8.0000

--- End Of Report ---

Agilent Technologies Page 1 of 1 Printed at: 10:28 AM on: 9/17/2020

Figure S4-2. ESI-MS spectrum of **4**

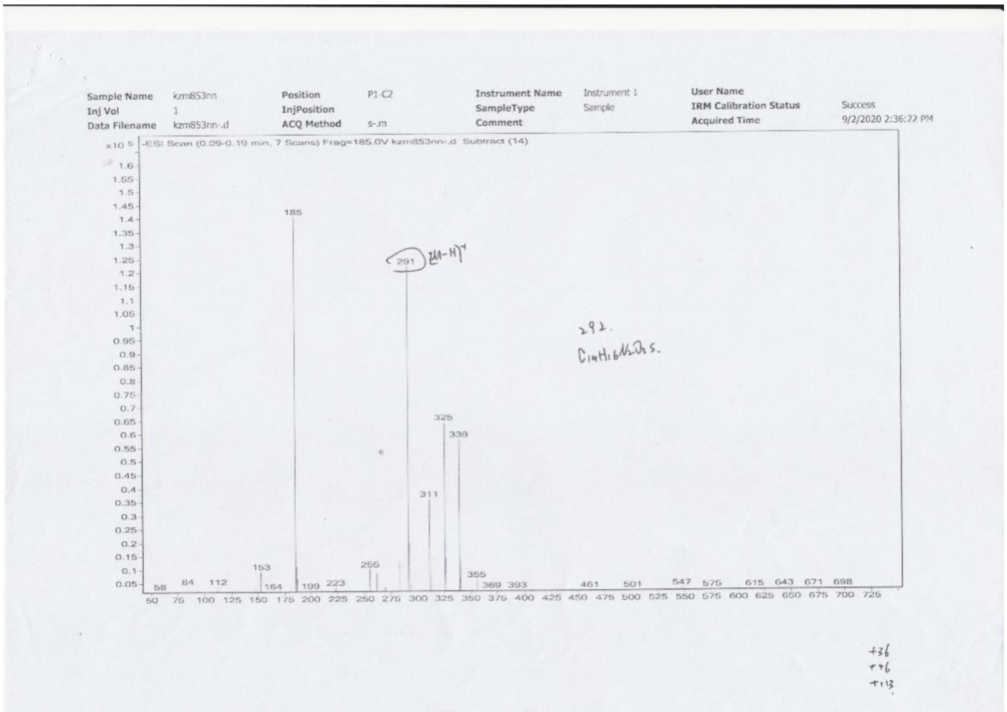

Figure S4-3 UV spectrum of **4** in MeOH

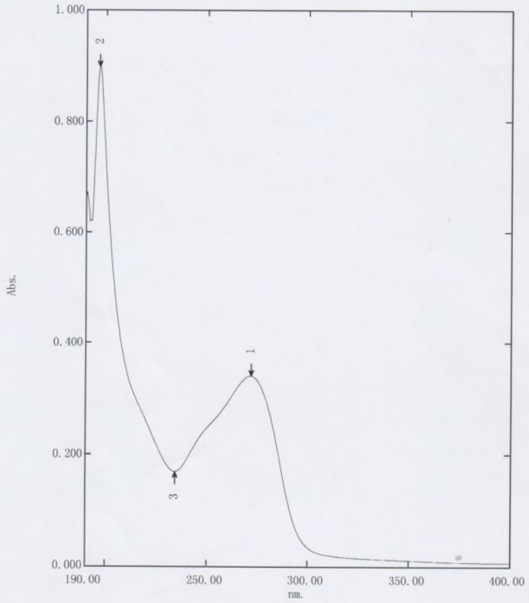

光谱峰值检测报告

2020/09/17 16:51:25

数据集: kzm853n - RawData

[测定属性]
波长范围(nm): 190.00 到 400.00
扫描速度: 中速
采样间隔: 0.5
自动采样间隔: 停用
扫描模式: 单个

[仪器属性]
仪器类型: UV-2700 系列
测定方式: 吸收值
狭缝宽: 5.0 nm
积分时间: 0.1 秒
光源转换波长: 323.0 nm
检测器单元: 直接
S/R 转换: 标准
阶梯校正: OFF

[附件属性]
附件: 无

[数据处理参数]
阈值: 0.0010000
点: 4
内插: 停用
平均: 停用

[样品准备属性]
重量:
体积:
稀释:
光程长: 10mm
附加信息: 样品浓度：0.0312毫克/毫升
溶剂：甲醇

No.	P/V	波长(nm)	Abs.	描述	
1	↑	271.50	0.341		3.50
2	↑	196.50	0.899		3.92
3	↓	234.50	0.169		3.20

页 1 / 1

Figure S4-4 [α] D data of **4**

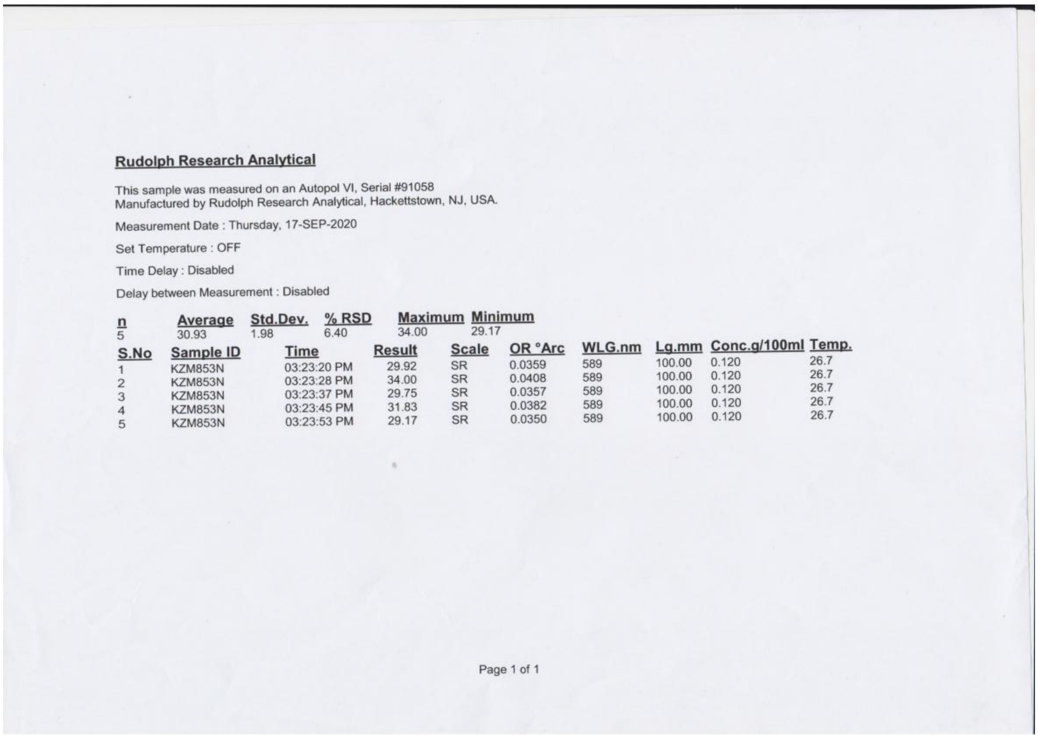

Rudolph Research Analytical

This sample was measured on an Autopol VI, Serial #91058
Manufactured by Rudolph Research Analytical, Hackettstown, NJ, USA.

Measurement Date : Thursday, 17-SEP-2020

Set Temperature : OFF

Time Delay : Disabled

Delay between Measurement : Disabled

n	Average	Std.Dev.	% RSD	Maximum	Minimum
5	30.93	1.98	6.40	34.00	29.17

S.No	Sample ID	Time	Result	Scale	OR °Arc	WLG.nm	Lg.mm	Conc.g/100ml	Temp.
1	KZM853N	03:23:20 PM	29.92	SR	0.0359	589	100.00	0.120	26.7
2	KZM853N	03:23:28 PM	34.00	SR	0.0408	589	100.00	0.120	26.7
3	KZM853N	03:23:37 PM	29.75	SR	0.0357	589	100.00	0.120	26.7
4	KZM853N	03:23:45 PM	31.83	SR	0.0382	589	100.00	0.120	26.7
5	KZM853N	03:23:53 PM	29.17	SR	0.0350	589	100.00	0.120	26.7

Page 1 of 1

Figure S4-5 ^{1}H NMR spectrum of **4** in $CDCl_3$ (600 MHz)

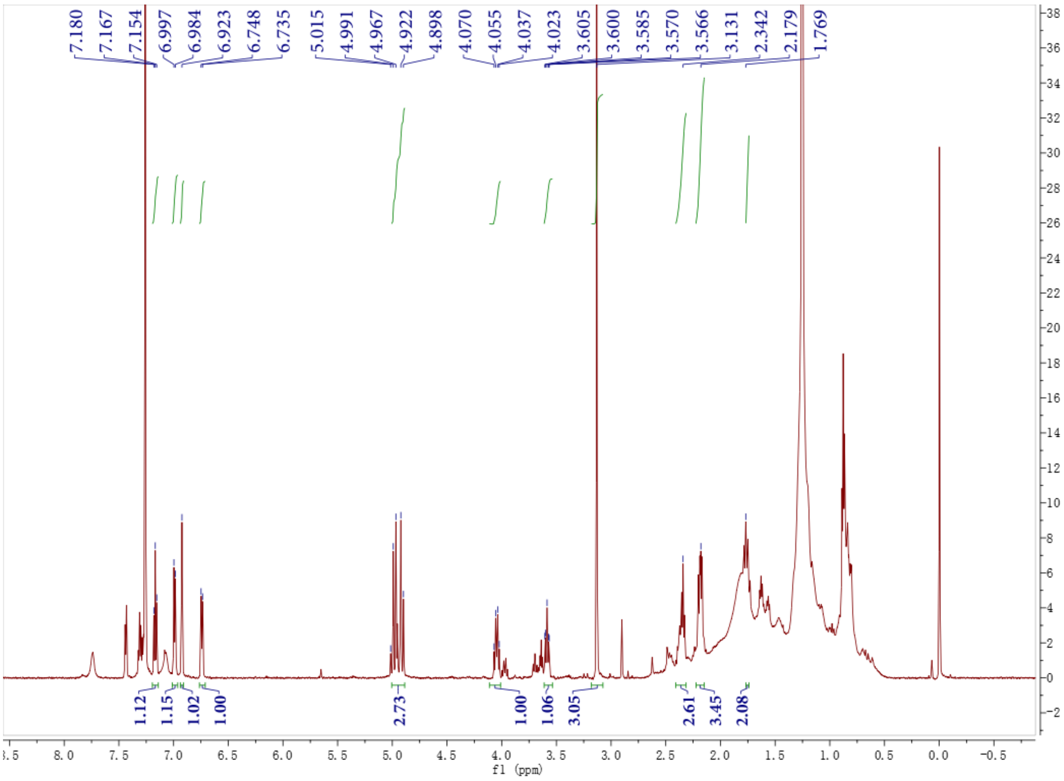

Figure S4-6 ^{13}C NMR spectrum of **4** in $CDCl_3$ (150 MHz)

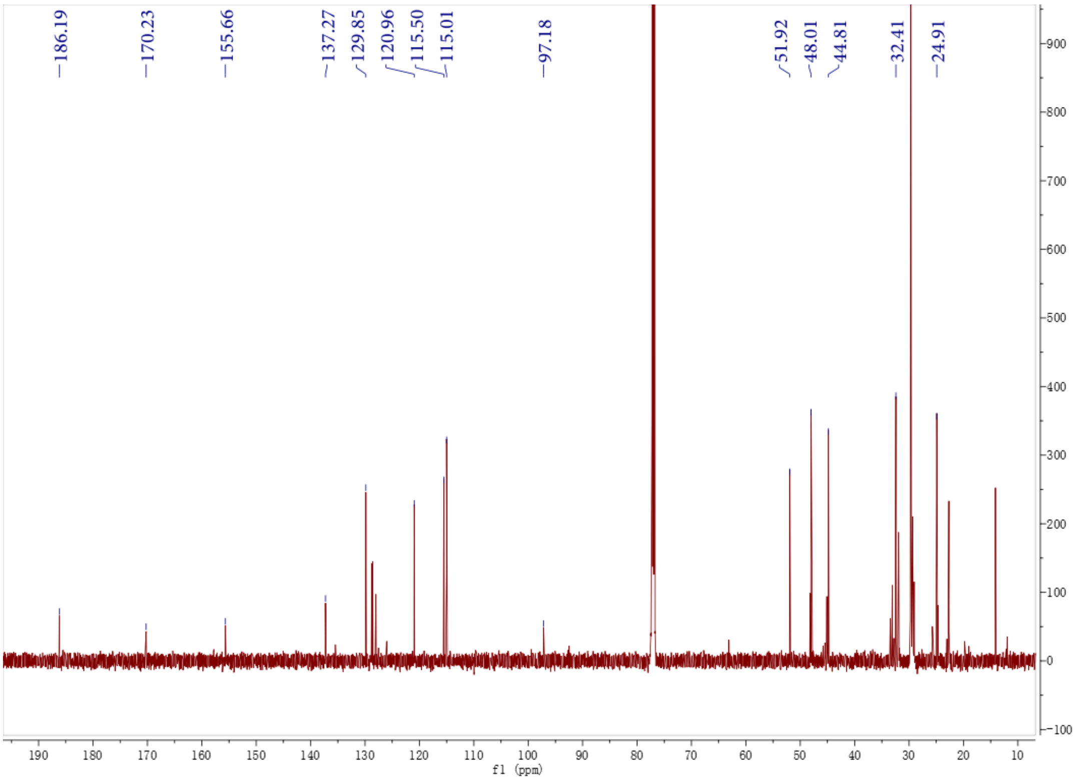

Figure S4-7 ^1H-^1H COSY spectrum of **4** in $CDCl_3$ (600 MHz)

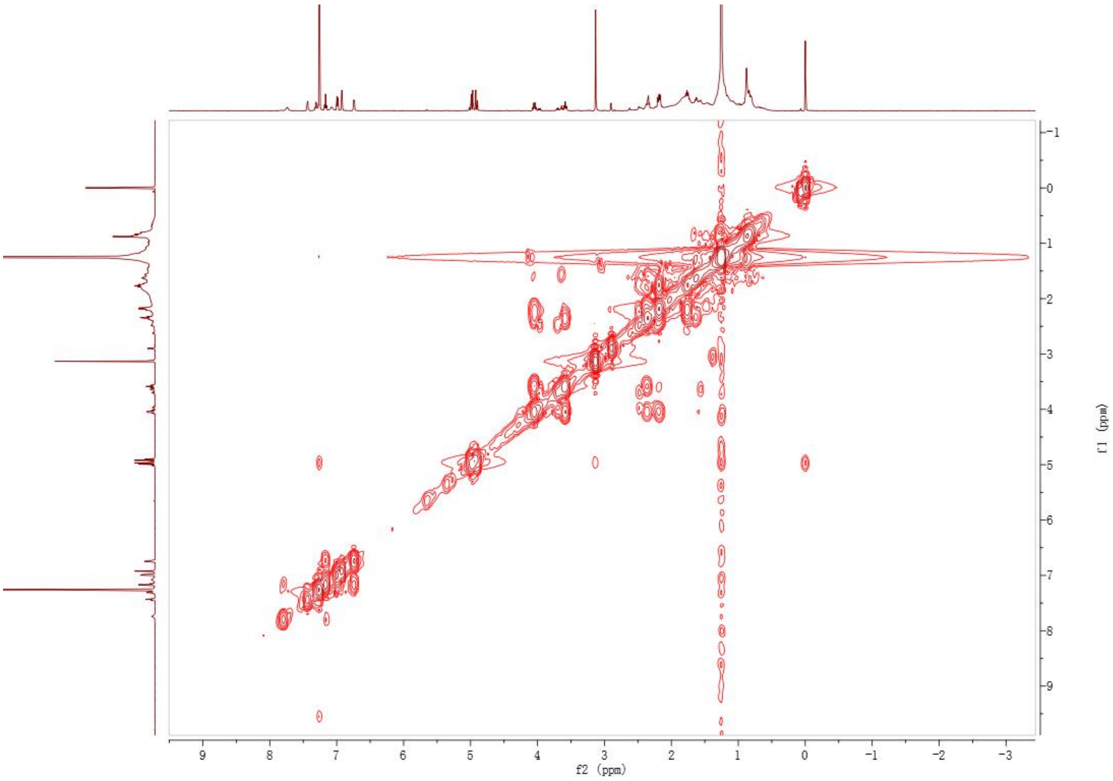

Figure S4-8 HSQC spectrum of **4** in $CDCl_3$ (600 MHz)

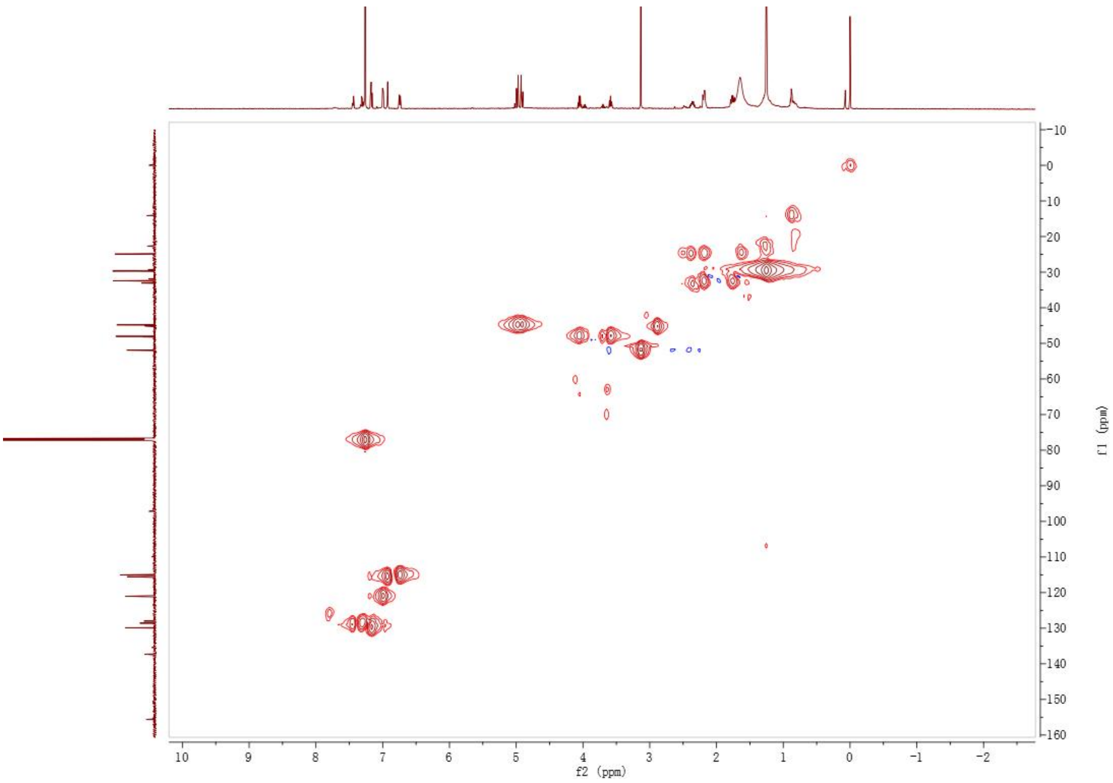

Figure S4-9 HMBC spectrum of **4** in $CDCl_3$ (600 MHz)

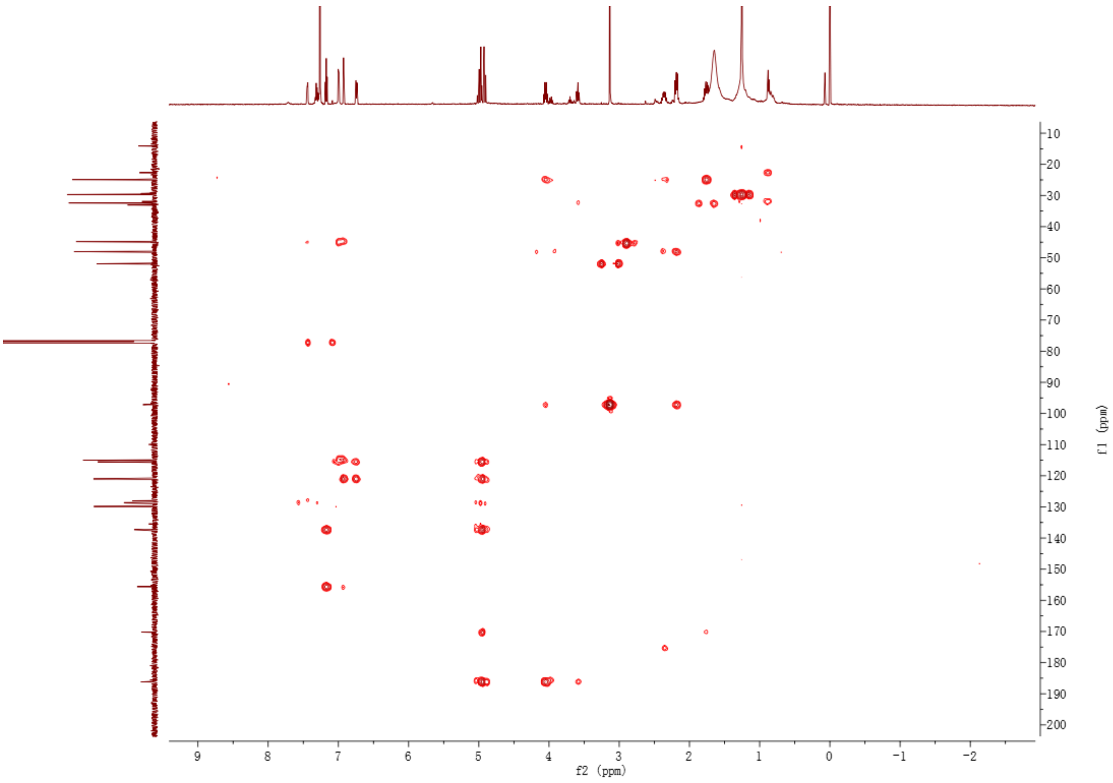

Figure S5-1. HR-ESI-MS spectrum of **5**

Qualitative Analysis Report

Data Filename	kmcr-11.d	Sample Name	kmcr-11
Sample Type	Sample	Position	P1-B6
Instrument Name	Instrument 1	User Name	
Acq Method	s-.m	Acquired Time	8/17/2020 1:19:40 PM
IRM Calibration Status	Success	DA Method	Default.m
Comment			

Sample Group		Info.
Acquisition SW Version	6200 series TOF/6500 series Q-TOF B.05.01 (B5125.2)	

User Spectra

Fragmentor Voltage	Collision Energy	Ionization Mode
135	0	ESI

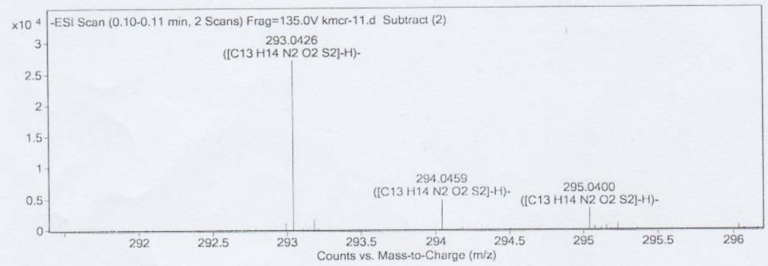

Peak List

m/z	z	Abund	Formula	Ion
89.0245		38950.94		
112.9854		22911.27		
241.051		14286.78		
269.0458	1	30657.91		
293.0426	1	27118.27	C13 H14 N2 O2 S2	(M-H)-
303.088	1	28193.79		
315.0881	1	43007.82		
333.0986	1	44211.24		
417.081	1	29548.55		
479.118	1	54096.62		

Formula Calculator Element Limits

Element	Min	Max
C	3	60
H	0	120
O	0	30
N	0	5
S	0	5
Cl	0	5

$C_{13}H_{16}N_2O_2S_2$

Formula Calculator Results

Formula	CalculatedMass	CalculatedMz	Mz	Diff. (mDa)	Diff. (ppm)	DBE
C13 H14 N2 O2 S2	294.0497	293.0424	293.0426	-0.20	-0.68	8.0000

--- End Of Report ---

Agilent Technologies   Page 1 of 1   Printed at: 3:44 PM on: 9/10/2020

Figure S5-2. ESI-MS spectrum of **5**

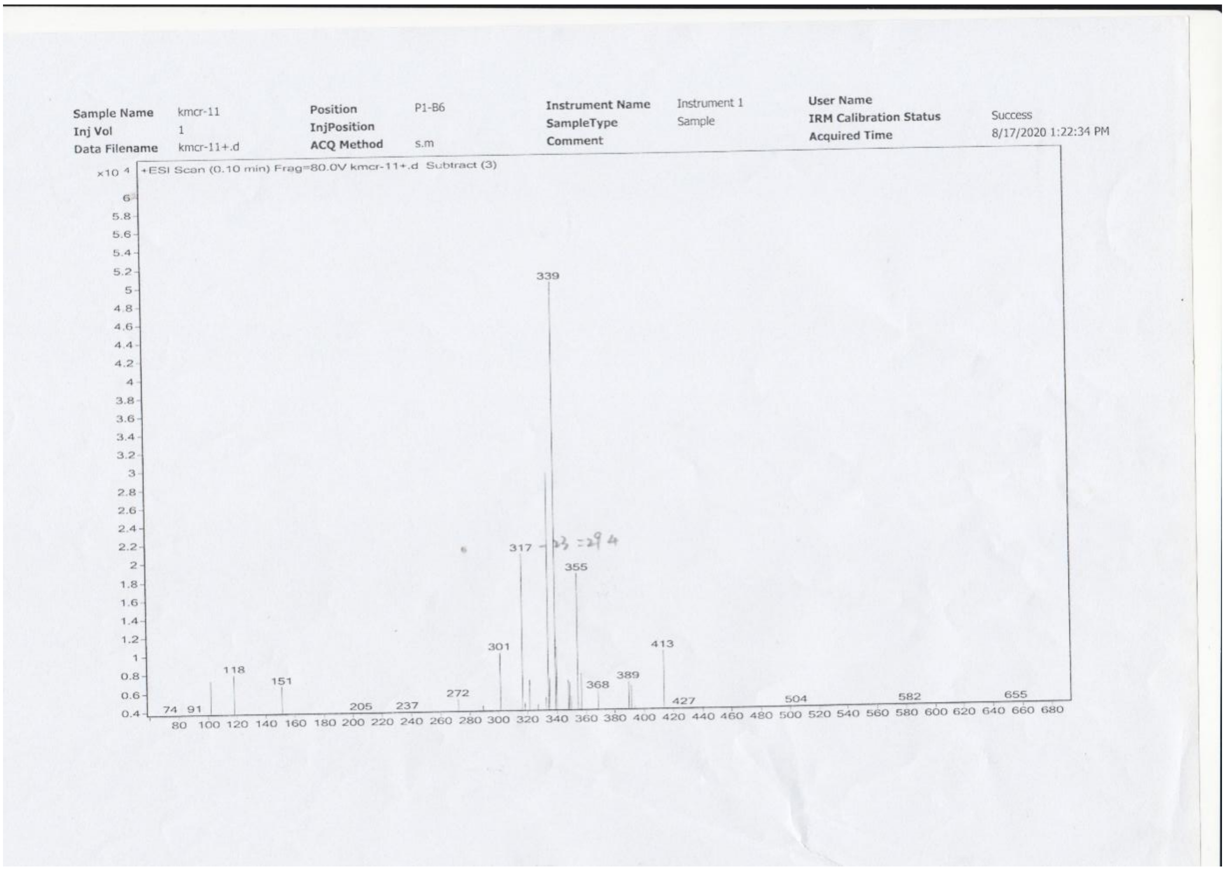

Figure S5-3 [α] D data of **5**

Rudolph Research Analytical

This sample was measured on an Autopol VI, Serial #91058
Manufactured by Rudolph Research Analytical, Hackettstown, NJ, USA.

Measurement Date : Thursday, 17-SEP-2020

Set Temperature : OFF

Time Delay : Disabled

Delay between Measurement : Disabled

n	Average	Std.Dev.	% RSD	Maximum	Minimum
5	108.38	1.04	0.95	109.88	107.38

S.No	Sample ID	Time	Result	Scale	OR °Arc	WLG.nm	Lg.mm	Conc.g/100ml	Temp.
1	KMCR-11	06:12:36 PM	108.25	SR	0.0866	589	100.00	0.080	26.0
2	KMCR-11	06:12:44 PM	107.50	SR	0.0860	589	100.00	0.080	26.0
3	KMCR-11	06:12:53 PM	107.38	SR	0.0859	589	100.00	0.080	26.0
4	KMCR-11	06:13:01 PM	108.88	SR	0.0871	589	100.00	0.080	26.1
5	KMCR-11	06:13:09 PM	109.88	SR	0.0879	589	100.00	0.080	26.1

Figure S5-4. CD and UV spectrum of **5** in MeOH

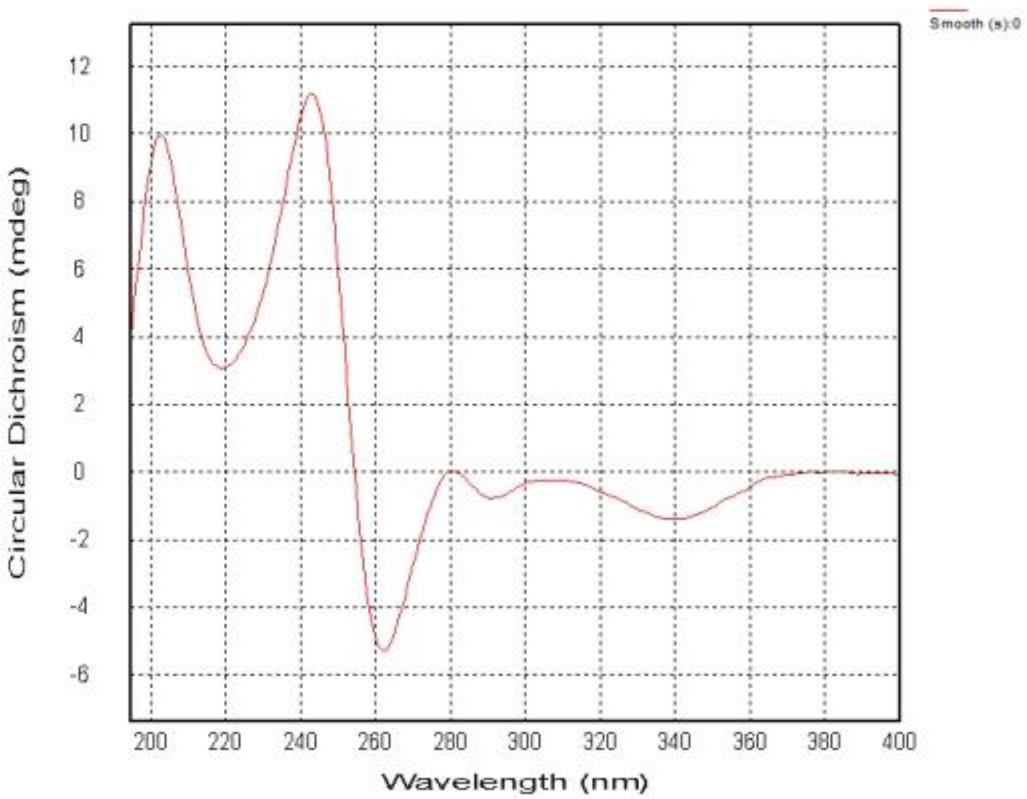

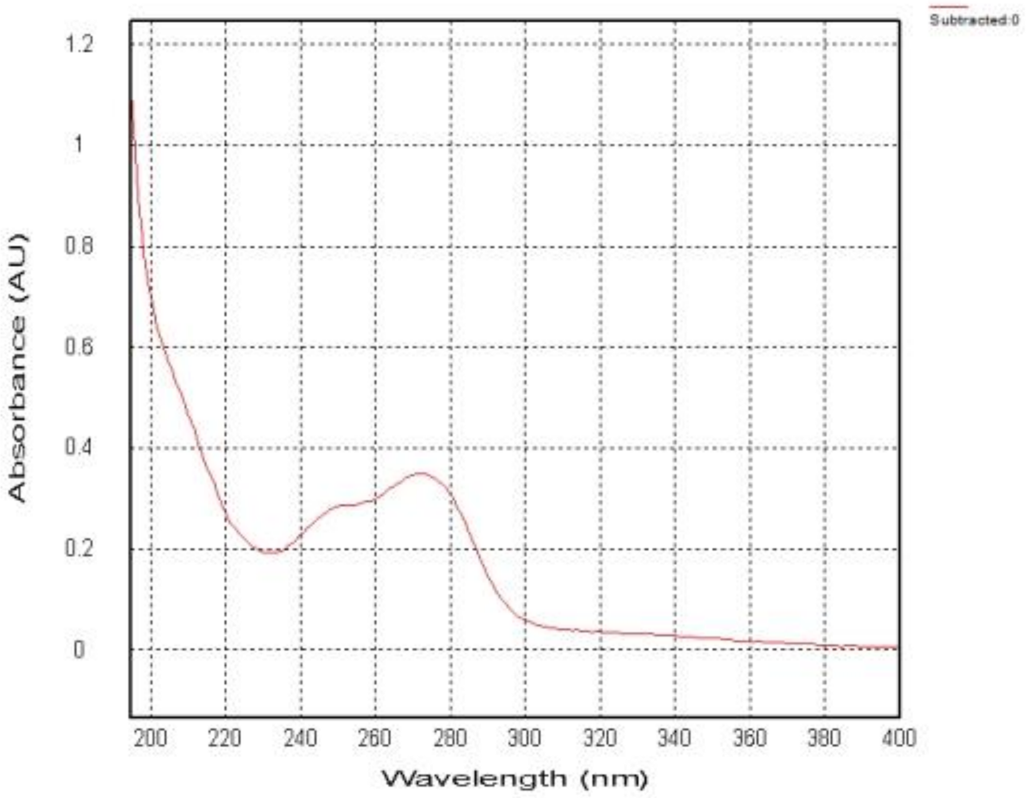

File: KMCR-11-1mm (195-400 nm) 20101316.dsx
ProBinaryX
Attributes :
- Time Stamp :Tue Oct 13 14:02:29 2020

- File ID : {B11F87C2-AC1E-49a4-A092-F2FC74DFBB25}
- Is CFR Compliant : false
- Original data has not been modified.
Remarks:
- User: CD
- Date: 2020/10/13
- Instrument: 0547
- DetectorType: LAAPD
- DichOS Calibration Correction Curve: 0547/2
- HV (CDDC channel): 0 v
- Time per point: 1 s
- Description: Sample 1
- Concentration: 0.0461mg/mL MeOH
- Pathlength: 1 mm
- Temperature: 20℃

Settings:
- Time-per-point: 1s (25us x 40000)
- SE
- Wavelength: 195nm - 400nm
- Step Size: 1nm
- Bandwidth: 1nm

Figure S5-5. ^{1}H NMR spectrum of **5** in $CDCl_3$ (600 MHz)

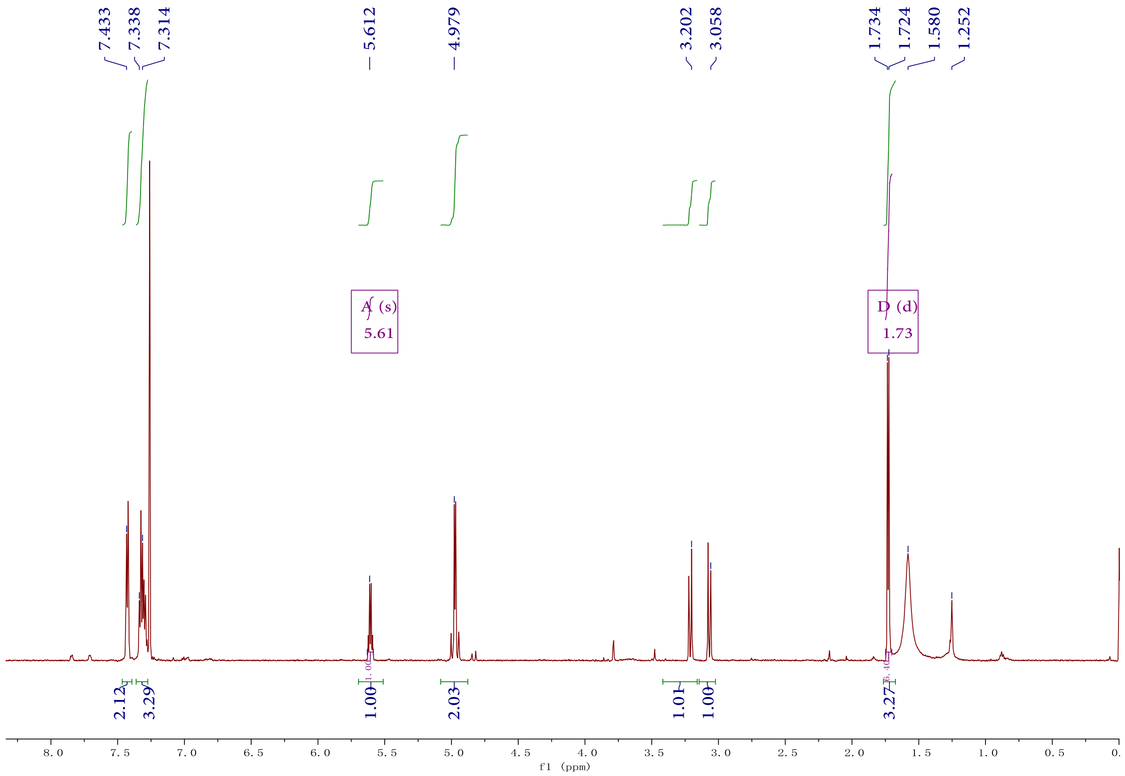

Figure S5-6. ^{13}C NMR spectrum of **5** in $CDCl_3$ (150 MHz)

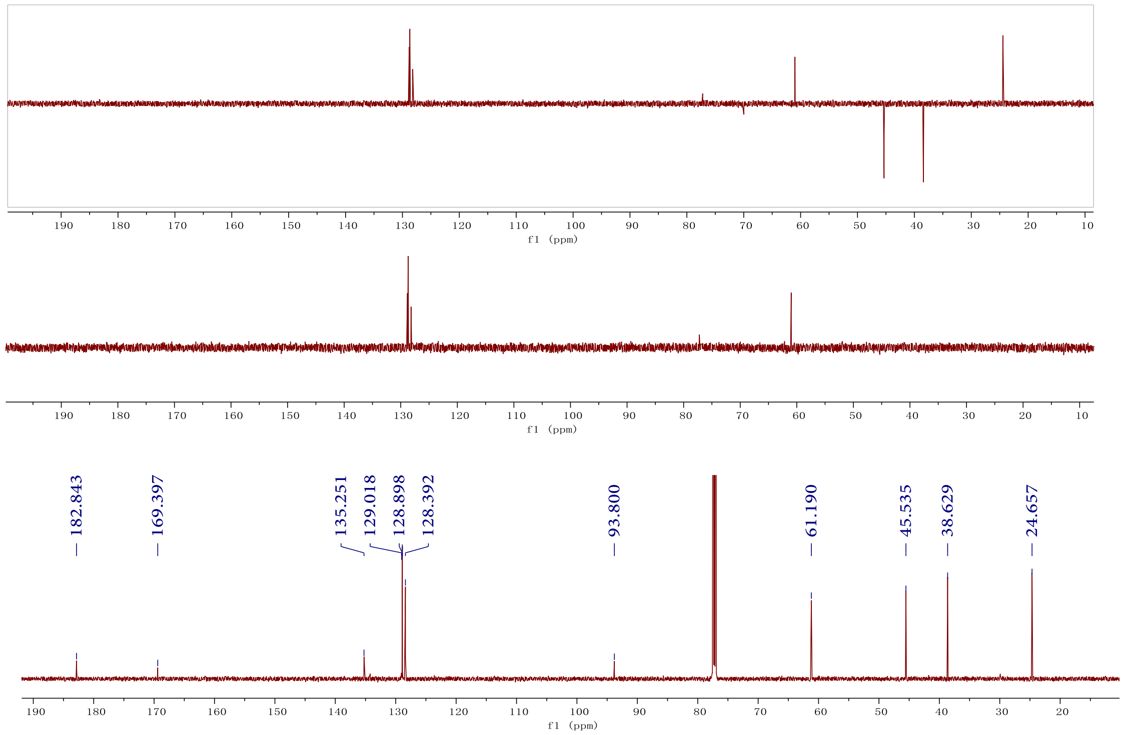

Figure S5-7. ^{1}H-^{1}H COSY spectrum of **5** in $CDCl_3$ (600 MHz)

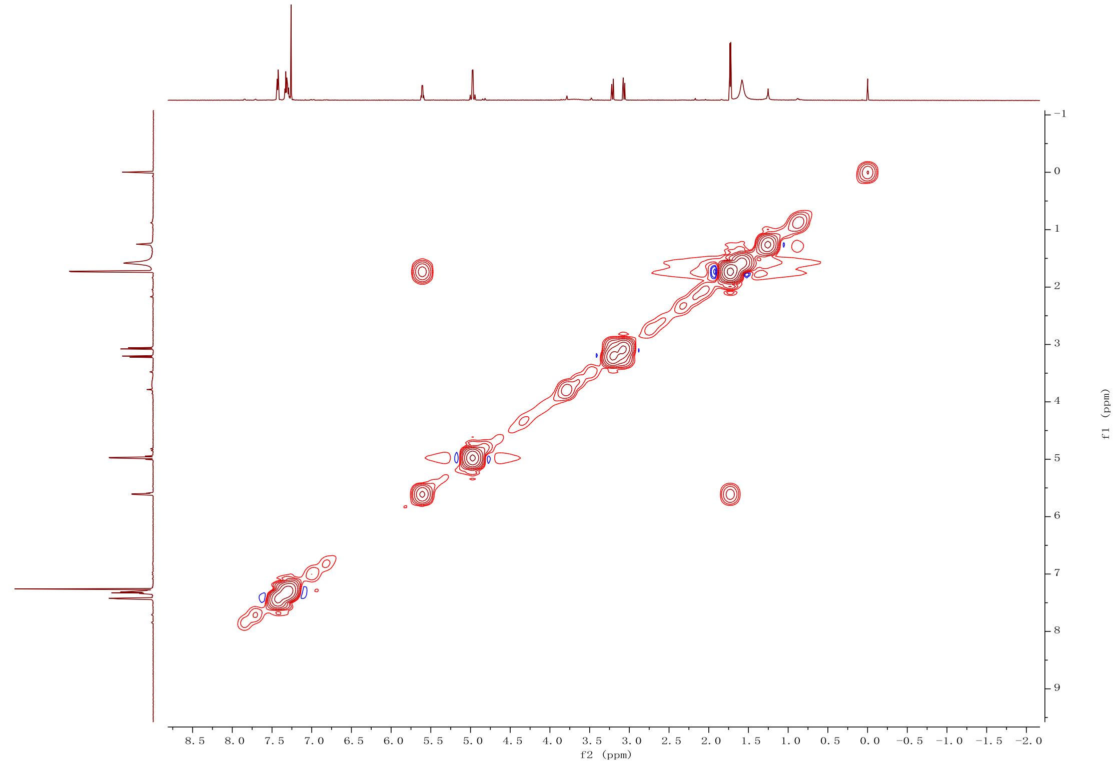

Figure S5-8. HSQC spectrum of **5** in $CDCl_3$ (600 MHz)

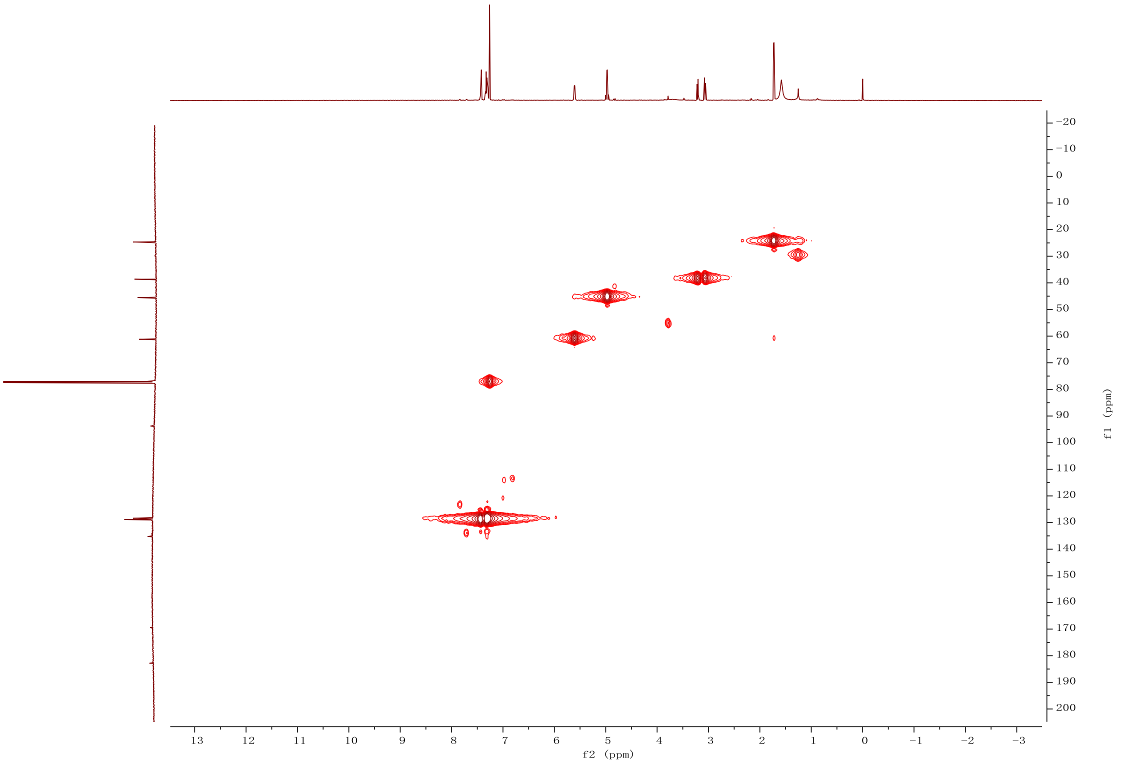

Figure S5-9. HMBC spectrum of **5** in $CDCl_3$ (600 MHz)

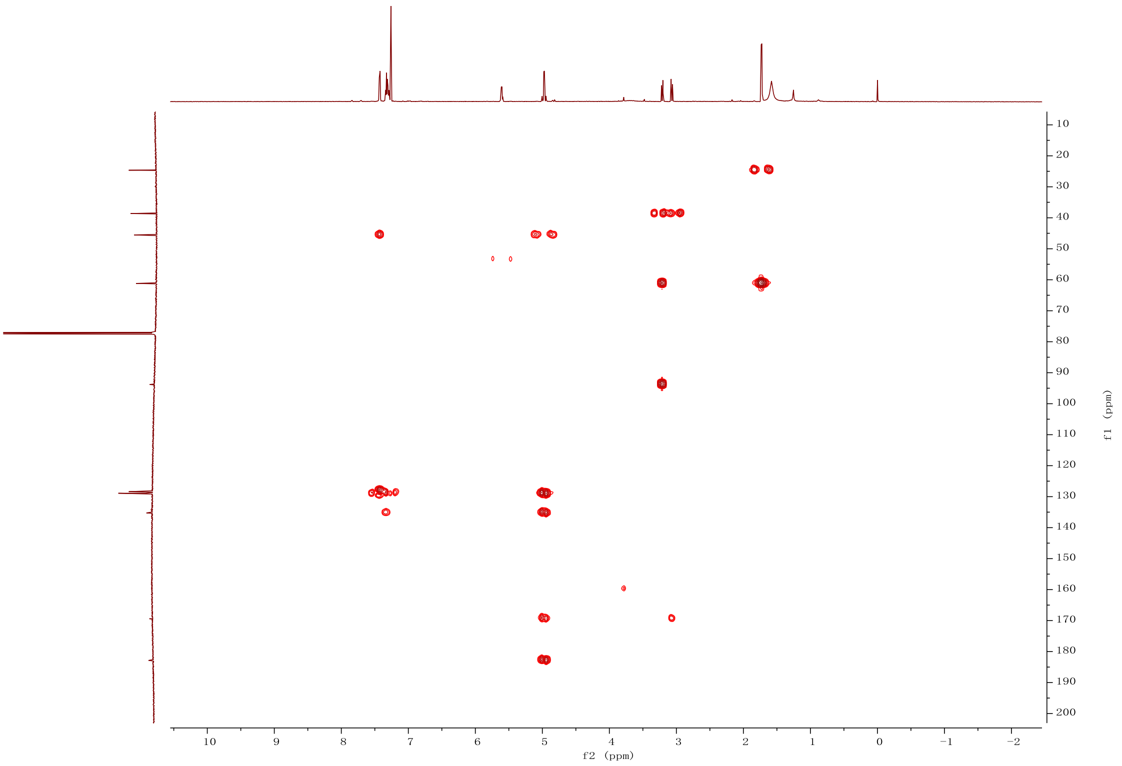

Figure S6 Neuroprotective activities of selected compounds

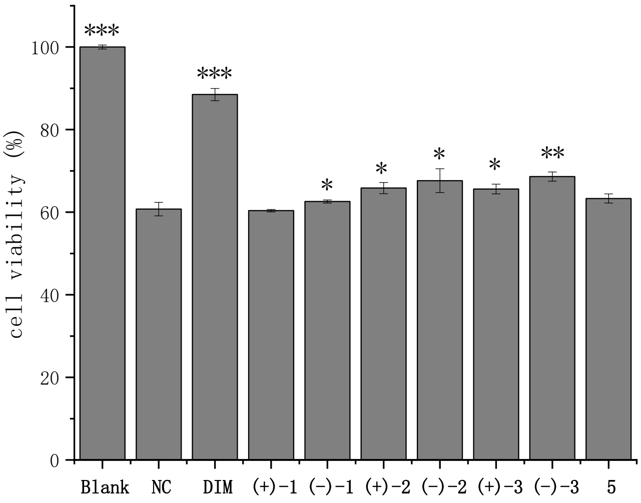

* P<0.05, ** p<0.01, and ***P< 0.001 vs NC group; NC: negative group; DIM: positive control group.
